# Electronic Structure and Chemical Bonding of MoX Molecules, where X = Li, Be, B, C, N, O, and F

**DOI:** 10.1021/acsomega.5c05197

**Published:** 2025-08-29

**Authors:** Alexandros Androutsopoulos, Demeter Tzeli

**Affiliations:** † Laboratory of Physical Chemistry, Department of Chemistry, 68993 National and Kapodistrian University of Athens Panepistimiopolis Zografou, Athens 15771, Greece; ‡ Theoretical and Physical Chemistry Institute, National Hellenic Research Foundation, 48 Vassileos Constantinou Ave, Athens 11635, Greece

## Abstract

In the present work, the ground state as well as some excited states of the MoX diatomic molecules, where X = Li, Be, B, C, N, O, and F, have been investigated to shed light on the nature of their chemical bonding. To this end, density functional theory, multireference configuration interaction and coupled-cluster methodologies have been employed in conjunction with aug-cc-pV5Z­(−PP) and aug-cc-pwCV5Z­(−PP) basis sets. Dissociation energies, dipole moments, and various spectroscopic constants are calculated with a view to studying the impact of the gradual increase of the number of valence electrons of X atom moving across the second period of the periodic table on the calculated properties of MoX. The Mo atom is a versatile atom forming different types of chemical bonds. The bonds formed in MoX range from a half bond (MoBe) to a quadruple bond (MoC), while all types of bonds are observed, including dative, covalent, and ionic. The corresponding dissociation energies range from 14.4 to 149.2 (152.5; CBS limit) kcal/mol at the C-MRCISD+Q and C-RCCSD­(T)/aug-cc-pV5Z­(−PP) levels. Finally, to evaluate the bond strength of the ground states, it was found that the dissociation energy per bond value is ∼ 25 kcal/mol for MoLi, MoBe and MoB, ∼ 40 kcal/mol for MoC and MoN, 61 kcal/mol for MoO and 111 kcal/mol for MoF which forms an ionic bond.

## Graphical abstract

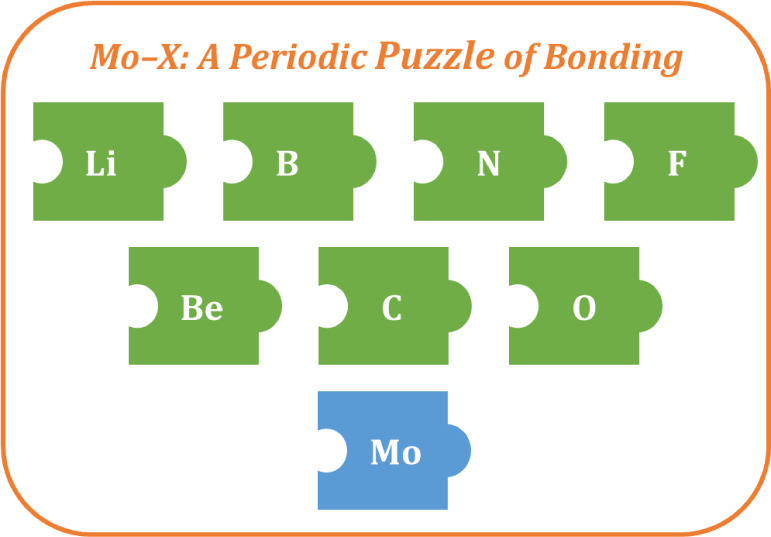

## 1 Introduction

It is well-known that transition metal compounds have been the subject of numerous studies, both theoretical and experimental, as they are involved in various research areas such as Catalysis,^1^ Surface Science,^2^ Organometallic Chemistry,^3^ Materials Science,^4^ Astrophysics,^5^ and Biology.^6^ In particular, the exploration of the electronic structure of molybdenum compounds is of considerable interest as they play a key role in nitrogen fixation and oxidation catalysis.^6,7^ Additionally, their importance in Materials Science is crucial due to applications in catalysis,^6–10^ energy conversion and storage,^8,10^ sensing,^10^ photonics,^10^ nanocomposites,^9,10^ and membranes.^10^ Consequently, a thorough insight into the simplest building blocks of such compounds, i.e., the diatomic molecules, serves as a step toward the investigation of more complex systems.

Molybdenum atom is unique since it has a 5s^1^4d^5^ electron configuration; that is, it can contribute all six valence electrons to the formation of chemical bonds.^11^ That “flexibility” is one of the reasons why it is involved in many different research areas. Thus, it is interesting to see how Mo interacts with the elements of the second period of the periodic table (Li–F) as well as how bond dissociation energy and other properties are affected from these interactions. The present study focuses on the electronic structure of the MoX species, where X = Li, Be, B, C, N, O, and F. Previously published experimental and theoretical data are reported below and summarized in Table 1.

**Table 1:** Previous Theoretical and Experimental Data on the Calculated States of the Present Work; Bond Lengths r_e_ (Å), Dissociation Energies D_e_ (D_0_) (kcal/mol) with respect to the Ground State Products, Vibrational Frequencies ω_e_ (cm^–1^), and Anharmonic Corrections ω_e_x_e_ (cm^–1^)

Molecule	Method	r_e_	D_e_(D_0_)	ω_e_	ω_e_x_e_
**MoB(** *X* ^**6**^ **Π)**	CASPT2^a^	1.968		664	
	B3LYP^b^	1.973	51.8	654.9	
**MoC (** *X* ^**3**^ **Σ** ^**–**^ **)**	Mass spectrometry^c^		(114.2 ± 3.8)		
	MRCISD^d^	1.688	119.3 (118.5)	997	
	R2PI spectroscopy^e^	1.6760			
	DF spectroscopy^f^	1.6877		1008.3	3.3
	MRCISD + Q/RECP^g^	1.717	102.4	968	
	RCCSD (T)/AVQZ – PP^h^	1.660			
	BP86/QZ4P^i^	1.667		1034.7	
	C – MRCISD + Q[CBS]^j^	1.671	118.3(116.7)	1038.3	6.76
	R2PI spectroscopy^k^		(118.4 ± 0.07)		
**MoN (Χ** ^**4**^ **Σ** ^**–**^ **)**	BP86/QZ4P^i^	1.634		1068.6	
	MI spectroscopy^l^			1040	
	GVB RCI^m^	1.603	94.0	1100.4	
	MRCISD^n^	1.636	119.2	1109	
	MRCI + Q/5Z^o^	1.640		1046	5.3
**MoO (X** ^**5**^ **Π)**	MI spectroscopy^l^			893.5	
	MRCISD + Q (SOC)^p^	1.707		880.5	
	VMI spectroscopy^q^		(125.9 ± 0.4)		
	R2PI spectroscopy^r^		(124.85)		
	LIF and SVL spectrosc.^s^			918.3	1.6
	LIF and SVL spectrosc.^t^	1.7129		918.8	2.0
	SDCI^u^	1.71	84.6	1035	
	MCPF/RECP^v^	1.709	(93.2)	910.0	
	LSD^w^	1.735	151.0	907	
	PIE spectroscopy^x^		(125.4)		
	LDA/RC^y^		(142.3)		
	B3LYP/LANL2DZ^z^	1.750		902	
	BP86/QZ4P[Table-fn tbl1fn27]	1.708		906.6	
**MoF (X** ^**6**^ **Σ** ^**+**^ **)**	Mass spectrometry[Table-fn tbl1fn28]		(110.3 ± 2.2)		
	MCPF[Table-fn tbl1fn29]	2.00[Table-fn tbl1fn29]	100.4		
	B3LYP[Table-fn tbl1fn30]	1.935	107.3	597	
	B3LYP[Table-fn tbl1fn31]	1.898	(113.9)	620	

a CASSCF//CASPT2/[8s7p5d3f2g/_Mo_5s4p3d2f/_B_]; ref. 12.

b B3LYP/aug-cc-pVQZ­(−PP); ref. 13.

c High temperature mass spectrometry; ref. 14.

d MRCISD/RC/[10s8p5d1f/_Mo_4s3p1d/_C_]; ref. 15

e r_0_ value, R2PI spectroscopy; ref. 16

f DF spectroscopy; ref. 18

g MRCISD+Q/RECP/[5s3p3d1f/_Mo_ 3s3p1d/_C_]; ref. 19

h CCSD­(T)/aug-cc-pwCVQZ­(−PP); ref. 19

i DFT/BP86/QZ4P; ref. 28

j Complete basis set limit (CBS) C-MRCISD+Q/aug-cc-pwCV*n*Z­(−PP), *n* = 2 – 5; ref. 23

k R2PI spectroscopy; ref. 23

l Matrix Isolation spectroscopy; ref. 24

m GVB RCI/[3s4p2d/_Mo_3s2p/_N_]; ref. 25

n MRCISD/[11s9p6d2f/_Mo_ 4s3p1d/_N_]; ref. 27

o MRCI+Q/5Z; ref. 29

p MRCISD+Q/SOC/[7s6p4d2f1g/_Mo_ 4s3p2d1f/_O_]; ref. 30

q VMI spectroscopy; ref. 31

r R2PI spectroscopy; ref. 32

s LIF and SVL spectroscopy; ref. 33

t LIF and SVL spectroscopy; ref. 34

u SDCI/(17s13p9d1f/_Mo_ 9s6p1d/_O_); ref. 35

v MCPF/RECP; ref. 36

w LSD; ref. 37

x PIE spectroscopy and MATI; ref. 41

y DFT/LDA/RC; ref. 41

z TDDFT/B3LYP/LANL2DZ; ref. 42

aa DFT/BP86/QZ4P; ref. 28

ab High temperature mass spectrometry; ref. 43

ac MCPF/[7s6p4d1f/_Mo_3s2p/_F_]; ref. 44

ad B3LYP/6–311++G­(df)-Stuttgart-Dresden; ref. 45

ae B3LYP/def2tzvpp; ref. 46

**MoB:** There are two theoretical studies on MoB. Borin and Gobbo^12^ have investigated the electronic structure and spectroscopic properties of the low-lying electronic states of MoB and MoB^+^ by implementing the CASSCF//CASPT2 method in conjunction with the Q-ζ ANO-RCC basis sets. In 2023, our group^13^ studied the second and third-row MBs via DFT calculations using the B3LYP, TPSSh, and MN15 functionals using the aug-cc-pVQZ­(−PP) basis set, in some cases, via multireference methods to compare the bonding and the properties of the MB molecules along the three rows. Our results are in agreement with the study of Borin and Gobbo,^12^ see Table 1.

**MoC:** The first study of MoC was carried out in 1981 by Gupta and Gingerich,^14^ as they measured the molecule’s bond dissociation energy via a Knudsen effusion mass spectroscopic study In that work, it was found that D_0_ = 4.95 ± 0.17 eV. Sixteen years later, Shim and Gingerich^15^ calculated some low-lying states via CASSCF and MRCI calculations, while relativistic corrections were also included. In 1998, Brugh et al.^16^ found that the ground state is the Ω = 0^+^ spin–orbit component of the ^3^Σ^–^ state with r_0_ = 1.6760 Å via optical spectroscopy. The following year, Li et al. measured the electron affinity of the MoC molecule via photoelectron spectroscopy and identified several excited electronic states.^17^ In 2001, DaBell et al.^18^ measured the term energies and vibrational frequencies of the ^3^Δ_2_ and ^1^Δ_2_ states via dispersed fluorescence spectroscopy. Computationally, in 2006, Denis and Balasubramanian^19^ calculated the potential energy curves and spectroscopic constants of the ground and 29 low-lying excited states of MoC employing the CASSCF and MRCISD methodologies, while Stevens et al.^20^ calculated the ground state of MoC using the DFT­(PB86) method. In 2007, the Steimle group measured the dipole moment of MoC for the v = 0 levels of *X* ^3^Σ^–^ and [18.6]^3^Π_1_ electronic states via high-resolution Stark spectroscopy;^21^ the notation [18.6] indicate that the state has been observed about 18.6 × 10^3^ cm^–1^ above the ground. In 2015, Liu et al. studied the MoC^–^ anion via a combined theoretical and experimental study.^22^ Recently, our group investigated the low-lying states of MoC, i.e., *X* ^3^Σ^–^, *A* ^3^Δ, *a* ^1^Γ, *c* ^3^Δ, *d* ^1^Σ^+^, and e^5^II, using multireference configuration interaction methodologies along with a series of basis sets to determine the basis set limit of all calculated properties.^23^ Relativistic effects and spin–orbit interactions were considered, while the ground state dissociation energy was measured precisely using resonant two-photon ionization (R2PI) spectroscopy.^23^ On the theoretical side, the dissociation energy D_e_(D_0_) with respect to the ground state products was extrapolated to the basis set limit, while the correction for scalar relativistic effects has also been considered. The final value of 5.13(5.06) eV is in excellent agreement with our measured D_0_value of 5.136(5) eV. Moreover, it was found that five electronic states, *X* ^3^Σ^–^,*A* ^3^Δ, a^1^Γ, c^1^Δ, *d* ^1^Σ^+^, possess a quadruple bond (σ^2^σ^2^π^2^π^2^) character; the calculated dissociation energies with respect to the adiabatic products range from 6.22 to 7.23 eV.^23^

**MoN:** It has been studied both experimentally and theoretically. In 1979, Bates and Gruen applied matrix isolation spectroscopy to investigate the molecule’s optical spectra.^24^ The ground state of Mo^14^N was identified as *X* ^4^Σ^–^ in an Ar matrix. In 1983, Allison and Goddard investigated the lower states of MoN at the GVB RCI/[3s4p2d/Mo 3s2p/N] level of theory^25^ and they concluded that the ground state of MoN, *X* ^4^Σ^–^, has a triple bond. In 1993, Fletcher et al. performed a high resolution optical spectroscopic study of MoN.^26^ Through that work, the permanent electric dipole moment of 3.38(7) D of the ground state, *X* ^4^Σ^–^, has been accurately determined. In 1999, Shim and Gingerich investigated the low-lying electronic states of MoN via all electron *abinitio* CASSCF as well as elaborate MRCI calculations.^27^ In 2006, Stevens et al.^28^ calculated the equilibrium bond length, dipole moment, and harmonic vibrational frequency of the ground state of MoN using flexible basis sets comprised of Slater type functions and a series of exchange correlation functionals. Finally, in 2024, White et al. investigated the interaction between N_2_ and Mo to provide an insight into nitrogen fixation.^29^

**MoO:** It is the most studied diatomic molecule among the MoX series. A brief description of the MoO studies before 2014 can be found in the study of Harms et al.,^30^ who analyzed the (0,0) band of the *c* ^3^Π_1_ – α Σ0+−3 transition based upon rotational analysis and *abinitio* calculations.^30^ In 2017, Couper et al.^31^ applied velocity map imaging (VMI) spectroscopy to MoO to determine the molecule’s ground state dissociation energy, D_0_ = 125.9 ± 0.4 kcal/mol in excellent agreement with the D_0_ value of 124.8 kcal/mol which obtained in 2020 by Sorensen et al.^32^ through the implementation of resonant two photon ionization (R2PI) spectroscopy and the predissociation threshold method. In 2021, Zhang et al.^33^ investigated the laser-induced fluorescence (LIF) excitation spectra and single vibronic level (SVL) emission spectra of the jet-cooled MoO molecule in the range of 16500–23200 cm^–1^. Totally 11 rotationally resolved excitation spectra were observed, and the rotational constants in 10 upper excited states and 4 lower spin–orbit components of the ground state, *X* ^5^Π, were obtained. A year later, Zhang et al.^34^ investigated four isotope-and rotationally resolved absorption bands of the MoO molecule using LIF excitation and SVL emission spectroscopy. More specifically, they concluded that all bands originate from a common lower state *X* ^5^Π_–1_ (v́́= 0) to upper states [14.11]­2, [14.15]­2, [14.24]­2 and [15.04]­2 while the rotational constants of the lower and the upper states for the seven natural ^i^MoO isotopologues were obtained. Concerning the theoretical work on MoO, three *abinitio* ^30,35,36^ and seven DFT^28,37–42^ articles have been published. The first *abinitio* study was published by Bauschlicher et al.^35^ in 1985. In that study they employed the SCF/CASSCF-SDCI approach around equilibrium for four states of MoO, i.e., two quintets (^5^Π, ^5^Π^+^) and two septets (^7^Π, ^7^Π^+^). In 1989, Langhoff et al.^36^ investigated the ground state and five low-lying excited states (^3^Δ, ^5^Π^+^, ^5^Δ, ^7^Π^+^, ^7^Π) of MoO by employing the MCPF and CASSCF/MRCI methods using pseudopotentials. In 2014, Harms et al.^30^ calculated seven singlet, 13 triplet, seven quintet and two septet states at the MRCISD+Q/VQZ level of theory to support the assignment of the transition they observed in their work. The first DFT study was published by Broclawik and Salahub^37^ in 1992. In the following years, a series of publications have been reported by the aforementioned authors employing the DFT methodologies.^38–40^ In 1998, Loock et al.^41^ investigated the properties of the low-lying excited states of MoO and MoO^+^ molecules employing a series of DFT calculations. The ground state dissociation energy including the first-order relativistic corrections calculated at 142.3 kcal/mol. In 2001, Broclawik and Borowski^42^ performed spin-unrestricted, time-dependent density functional theory (TDDFT) calculations for seven quintet states *X* ^5^Π, *A* ^5^Σ^+^, ^5^Σ^–^, *A*′^5^Δ, *B* ^5^Π, *B*′^5^Π, ^5^Φ) and two septet (^7^Π, ^7^Π^+^) states of MoO. Finally, in 2006, Stevens et al.^28^ calculated the equilibrium bond length, dipole moment, and harmonic vibrational frequency of the ground state, *X* ^5^Π, of MoO using flexible basis sets comprised of Slater type functions and a series of exchange correlation functionals.

**MoF:** In 1976, Hildenbrand^43^ carried out a thermochemical study of the gaseous molybdenum fluorides and found that the ground state dissociation energy, D_0_, was equal to 110.3 ± 2.2 kcal/mol. In 1993, Siegbahn^44^ performed a series of correlated calculations for the diatomic second row transition metal hydrides, fluorides, and chlorides. The ground state of MoF was deduced to be of *X* ^6^Σ^+^ symmetry with r_e_ = 2.00 Å and D_e_ = 100.4 kcal/mol. In 2007, Cheng et al.^45^ investigated the bond distances, vibrational frequencies, dipole moments, dissociation energies, electron affinities, and ionization potentials of MX (XM = Y–Cd, X = F, Cl, Br, I) molecules in neutral, positively, and negatively charged ions by employing the density functional method. In 2022, Sakr et al.^46^ performed a combined density functional theory and experimental study of the molecular molybdenum fluorides, MoF to MoF_6_.

In the present work, we investigated the ground state as well as some excited states of MoX diatomic species (X = Li- F), to shed light on the nature of their chemical bonding. More specifically, we have calculated the spectroscopic constants and potential energy curves for the states in question employing multireference configuration interaction and coupled-cluster methodologies. In addition, we studied how all calculated data are affected as the X atom changes across the second row of the periodic table, i.e., from Li to F. Molybdenum provides a solid background for this kind of study, in the sense that it is quite flexible when it comes to interacting with other elements, as it consists of six unpaired valence electrons.

## 2 Computational Details

As first step, the ground states of MoX were calculated at the density functional theory level using three functionals i.e., B3LYP,^47^ MN15,^48^ and TPSSh,^49^ in conjunction with the aug-cc-pVQZ-PP and aug-cc-pVQZ basis sets for Mo^50^ and X (Li–F) atoms,^51^ respectively. These results were compared with the ones of multireference and coupled-cluster calculations (more details below) in order to determine which functional most closely agrees with these high-level methods.

The ground state as well as some excited states of MoX were investigated via multireference and coupled-cluster methods using correlation consistent basis sets of quintuple-ζ quality, i.e., **Mo:** aug-cc-pV5Z-PP^50^ contracted to [8s8p7d5f4g3h2i] and aug-cc-pwCV5Z-PP,^50^ contracted to [10s10p9d6f5g4h3i] and **X:** aug-cc-pV5Z^51^ contracted to [7s6p5d4f3g2h], and aug-cc-pwCV5Z^51,52^ contracted to [11s10p8d6f4g2h]. For Mo, the basis sets mentioned above employ accurate core relativistic pseudopotentials for the 1s^2^2s^2^2p^6^3s^2^3p^6^electrons and treat the 4s^2^4p^6^(5s4d)^6^ electrons explicitly. Regarding MoLi and MoBe, a series of complete active space self-consistent field (CASSCF) calculations was performed to determine their ground state symmetries.

The present work is based on three methods of calculation: CASSCF, CASSCF + single + double replacements (CASSCF + 1 + 2 = MRCISD), and restricted coupled-cluster + singles + doubles + perturbative connected triples [RCCSD­(T)].^53–55^ Due to convergence issues, the ground state of MoC has not been studied via the coupled-cluster method.

For MoLi, MoBe, MoB, MoC and MoN, the CASSCF reference wave functions are built by distributing 7 valence electrons [Mo (4d^5^5s^1^) + Li (2s^1^)] up to 11 valence electrons [Mo (4d^5^5s^1^) + N (2s^2^2p^3^)] to ten orbital functions, one 5s and five 4d’s on Mo + one 2s and three 2p’s on Li, Be, B, C and N. In the case of MoO, 10 electrons [Mo (4d^5^5s^1^) + O (2p^4^)] are distributed to nine orbital functions, one 5s and five 4d’s on Mo + three 2p’s on O; that is, the 2s^2^ electrons of oxygen remain doubly occupied. Then, as a next step, the MRCISD method was employed. To take core correlation effects into account, the 4s^2^4p^6^ electrons of the metal atom and the 1s^2^ electrons of the X atoms have been included in the MRCISD space (C-MRCISD) using the weighted core–valence basis sets. For MoF, C-CASSCF calculations were carried out, where all 23 electrons [Mo (4s^2^4p^6^4d^5^5s^1^) + F (1s^2^2s^2^2p^5^)] are active electrons and are distributed to 15 orbital functions to ensure the correct energetic ordering of the molecular orbitals. Our reference spaces range from 303 (MoBe; *X* ^7^Π^+^) to 17130 (MoF; ^6^Π) configuration state functions (CSFs), with corresponding MRCI spaces ranging from about 4 × 10^9^ (MoBe; *X* ^7^Π^+^) to 6 × 10^10^ (MoF; ^6^Π) CSFs. For the MRCISD and C-MRCISD calculations, the internal contraction approach is applied.^56^ Finally, the Davidson correction + Q^57^ was incorporated into the MRCISD and C-MRCISD methodologies.

Two types of coupled-cluster calculations were carried out: (1) RCCSD­(T)/aug-cc-pV5Z­(−PP), which takes only the valence electrons into account [7 electrons (MoLi) – 13 electrons (MoF)], and (2) C-RCCSD­(T)/aug-cc-pwCV5Z­(−PP), where all electrons are correlated. In this level of theory, the weighted core–valence basis sets were used. More specifically, in the C-RCCSD­(T) calculations, 17 electrons [Mo (4s^2^4p^6^4d^5^5s^1^) + Li (1s^2^2s^1^)] up to 23 electrons [Mo (4s^2^4p^6^4d^5^5s^1^) + F (1s^2^2s^2^2p^5^)] are correlated, while the corresponding C-RCCSD­(T) space ranges from about 5 × 10^6^ to 9 × 10^6^ CSFs. To assess the accuracy of our C-RCCSD­(T) method, the T_1_ diagnostic was checked. In all cases, T_1_ was about 0.04 or less, which denotes the method’s accuracy.

The potential energy curves of all calculated states are obtained at the C-MRCISD+Q level. Bond distances, dissociation energies, and other spectroscopic constants are calculated at all levels of theory. For the multireference methodologies, dissociation energies have been calculated as D_e_ = E_∞_(MoX) – E_e_(MoX), where E_∞_(MoX) is the energy of the Mo-X molecule at r = 15 Å and E_e_(MoX) is the corresponding equilibrium energy. At the coupled cluster and DFT calculations, dissociation energies are calculated as D_e_ = [E­(Mo) + E­(X)] – E_e_(MoX), where E­(Mo) + E­(X) are the energies of the Mo and X atoms. A Dunham analysis^58^ is used to extract all spectroscopic constants. In each case, the bonding has been analyzed as it is represented through valence bond Lewis (vbL) diagrams. All calculations were done under C_2v_ symmetry constraints. DFT calculations were carried out via Gaussian16.^59^ Multireference and coupled-cluster calculations were performed using the MOLPRO^60^ suite of codes.

## 3 Results and Discussion

The potential energy curves of the ground state and some excited states of MoX species, where X = Li, Be, B, C, N, O, and F, have been calculated. Bond distances (r_e_), dissociation energies (D_e_), harmonic frequencies ω_e_), anharmonic corrections (*ω*_e_x_e_), and dipole moments (μ) calculated either as expectation values or via the finite field method have been obtained, see Table 2. The potential energy curves (PECs) of the ground state of all species are depicted in Figure 1 for reasons of comparison. Specifically, it is shown that the MoC molecule presents the largest D_e_ values, i.e., the deepest PEC, while the MoBe molecules has the smallest one. In what follows, each section encompasses the results obtained for each diatomic molecule. Additionally, we investigate the effect of the gradual increase of the number of valence electrons of X atom as we move across the second period of the periodic table on our calculated properties while the bonding schemes have been analyzed for all cases. Finally, we give some physical and chemical insights into the molecules and materials in which Mo atom is involved.

**Table 2:** Bond Distances r_e_ (Å), Adiabatic Dissociation Energies D_e_ (kcal/mol), Harmonic Frequencies ω_e_ (cm^–1^), Anharmonic Corrections ω_e_x_e_ (cm^–1^), Dipole Moments μ (D) and Relative Energies T_e_ (kcal/mol) of the Ground and Excited States of MoX Using the aug-Cc-pV5z­(−pp) and aug-Cc-pwCv5z­(−pp) Basis Sets

MoX	State	Methodology^a^	r_e_	D_e_ ^b^	ω_e_	ω_e_x_e_	μ_FF_ ^c^	⟨μ⟩^c^	T^e^
**MoLi**	X^6^Σ^+^	B3LYP	2.692	24.4	314.2			3.18	
		TPSSH	2.772	22.1	289.3			2.43	
		MN15	2.614	29.9	362.3			3.67	
		MRCISD	2.717	22.7	312.1	2.12	3.46	2.69	
		MRCISD+Q	2.708	24.0	316.8	2.11	3.63		
		RCCSD(T)	2.708	23.8	320.4	3.57	3.56		
		C-MRCISD	2.702	19.6	320.3	2.74	3.30	2.08	
		C-MRCISD+Q	2.677	22.2	331.8	2.65	3.74		
		C-RCCSD(T)	2.667	24.4	329.6	3.42	3.73		
**MoBe**	X^7^Σ^+^	B3LYP	2.692	24.4	314.2			3.18	
		TPSSH	2.454	23.4	371.8			1.52	
		MN15	2.406	19.2	340.7			0.97	
		MRCISD	2.481	11.4	330.7	6.26	1.41	0.94	
		MRCISD+Q	2.462	13.8	351.1	5.66	1.51		
		RCCSD(T)	2.481	13.5	340.3	8.22	1.28		
		C-MRCISD	2.474	8.5	344.1	7.38	1.38	0.76	
		C-MRCISD+Q	2.431	13.2	383.4	5.93	1.65		
		C-RCCSD(T)	2.452	14.4	349.4	11.63	1.24		
**MoB**	X^6^Π	B3LYP	1.973	51.8	654.9			2.22	
		TPSSH	1.982	53.9	645.8			2.42	
		MN15	1.941	56.8	709.9			1.99	
		MRCISD	1.990	43.3	653.1	6.55	2.43	2.29	
		MRCISD+Q	1.991	46.4	652.0	6.46	2.44		
		RCCSD(T)	1.979	45.2	671.0	7.46	2.46		
		C-MRCISD	1.978	42.3	677.1	5.53	2.43	2.19	
		C-MRCISD+Q	1.974	48.2	685.0	5.25	2.47		
		C-RCCSD(T)	1.959	47.2	698.2	8.64	2.31		
	A^6^Σ^+^	MRCISD	2.133	37.8	449.0	5.03	2.69	2.54	5.36
		MRCISD+Q	2.129	41.1	455.0	4.67	2.73		5.40
		RCCSD(T)	2.071	41.3	593.0	8.95	2.75		3.95
		C-MRCISD	2.112	36.1	513.0	6.41	2.70		5.83
		C-MRCISD+Q	2.101	42.1	535.9	5.54	2.80		6.07
		C-RCCSD(T)	2.104	41.8	489.4	7.11	2.64		5.39
**MoC**	X^3^Σ^–^	B3LYP	1.661	126.8	1070.1			3.24	
		TPSSH	1.668	137.3	1029.3			5.47	
		MN15	1.644	132.2	1116.8			3.05	
		MRCISD	1.679	142.9(110.7)	1027.5	7.21	5.96	6.00	
		MRCISD+Q	1.682	143.4(113.2)	1022.2	7.29	5.92		
		C-MRCISD	1.670	146.1(110.5)	1042.9	6.76	5.88	5.99	
		C-MRCISD+Q	1.673	149.2(115.0)	1038.3	6.76	5.83		
		C-MRCISD+Q[CBS]^d^	1.671	(118.3)					
		Expt.	1.6760^e^	(118.4 ± 0.07)^f^					
		Expt.	1.6877^g^		1008.3^g^	3.3^g^			
**MoN**	X^4^Σ^–^	B3LYP	1.622	126.0	1109.8			3.28	
		TPSSH	1.628	121.9	1088.0			3.27	
		MN15	1.605	132.4	1172.5			3.28	
		MRCISD	1.637	117.8	1066.1	6.42	3.02	2.71	
		MRCISD+Q	1.639	120.2	1061.0	6.55	3.10		
		RCCSD(T)	1.634	121.2	1087.0	7.10	3.16		
		C-MRCISD	1.632	118.6	1077.0	7.18	2.97	2.58	
		C-MRCISD+Q	1.635	123.7	1070.1	7.34	3.11		
		C-RCCSD(T)	1.627	122.6	1092.8	7.51	3.09		
**MoO**	X^5^Π	B3LYP	1.697	126.0	954.7			3.32	
		TPSSH	1.699	123.5	946.6			3.20	
		MN15	1.678	131.5	1008.0			3.39	
		MRCISD	1.714	111.9	940.0	5.51	3.29	2.94	
		MRCISD+Q	1.711	118.7	946.1	5.32	3.30		
		RCCSD(T)	1.707	121.9	962.0	5.02	3.22		
		C-MRCISD	1.708	109.0	942.4	5.36	3.32	2.77	
		C-MRCISD+Q	1.706	118.1	948.8	5.08	3.42		
		C-RCCSD(T)	1.696	122.9	944.8	6.79	3.15		
		Expt.^h^	1.7129		918.8	2.0			
	^5^Σ^–^	MRCISD	1.737	86.8	906.1	6.05	0.22	0.21	24.79
		MRCISD+Q	1.741	91.4	897.0	6.29	0.19		27.35
		RCCSD(T)	1.735	91.3	888.0	6.47	0.16		30.64
		C-MRCISD	1.729	84.3	899.4	5.36	0.21		
		C-MRCISD+Q	1.732	91.3	890.2	5.55	0.19		
		C-RCCSD(T)	1.726	91.4	892.9	6.88	0.13		
**MoF**	X^6^Σ^+^	B3LYP	1.903	114.6	617.7			3.37	
		TPSSH	1.895	113.8	630.9			3.17	
		MN15	1.885	118.7	645.3			3.42	
		RCCSD(T)	1.913	111.0	621.0	6.20	3.75		
		C-MRCISD	1.896	103.9	651.0	4.28	3.83	3.65	
		C-MRCISD+Q	1.898	108.5	647.4	4.31	3.87		
		C-RCCSD(T)	1.895	111.4	627.6	6.54	3.64		
	Π^6^	C-MRCISD	1.953	71.4	616.9	2.77	1.60		
		C-MRCISD+Q	1.951	76.0	618.3	2.74	1.48		
		C-RCCSD(T)	1.947	79.2	580.8	5.34	1.27		

a The weighted core basis set is used for the “C-” methods, i.e., the core–valence correlation has been considered.

b Dissociation energies with respect to the correlated fragments; the values in parentheses correspond to dissociation energies with respect to the ground state fragments when they are different from the correlated fragments.

c ⟨ μ ⟩ refers to expectation values and μ_FF_ to finite field values; the absolute values are given.

d Complete basis set (CBS) limit at the C-MRCISD+Q level using the aug-cc-pwCV*n*Z­(−PP) basis sets, *n* = 2 – 5; ref. 23

e r_0_ value, R2PI spectroscopy; ref. 16

f R2PI spectroscopy; ref. 23

g DF spectroscopy; ref. 18

h LIF and SVL spectroscopy; ref .^34^

**Figure 1:**
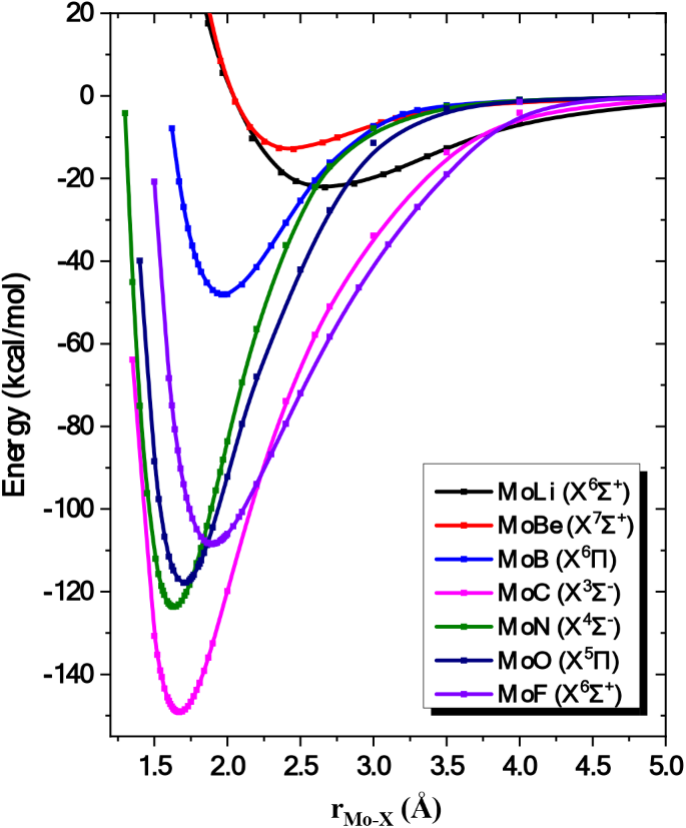
Potential energy curves of the ground states of MoX molecules at the MRCISD+Q/aug-cc-pV5Z­(−PP) level. All energies are referenced to the separated Mo and X atomic fragments.

### 3.1 MoLi

The interaction between the ground state fragments, Li (^2^S) and Mo (^7^S), gives rise to two electronic states of ^6^Σ^+^ and ^8^Σ^+^ symmetry. Since there are not any previous works on MoLi, exploratory CASSCF calculations were performed to determine the molecule’s ground state. In particular, the lowest states of A_1_ and A_2_ spatial symmetry corresponding to Σ^+^, Σ^–^ or Δ*s*tates as well as the lowest states of B_1_ and B_2_ spatial symmetry corresponding to Π or Φ states have been calculated within C_2v_ symmetry constraints for a series of spin symmetries, i.e., for doublets, quartets, sextets and octets. The obtained potential energy curves are depicted in Figure 2. It is found that the ground state is of ^6^Σ^+^ symmetry. As expected, the octet state is almost repulsive.

**Figure 2:**
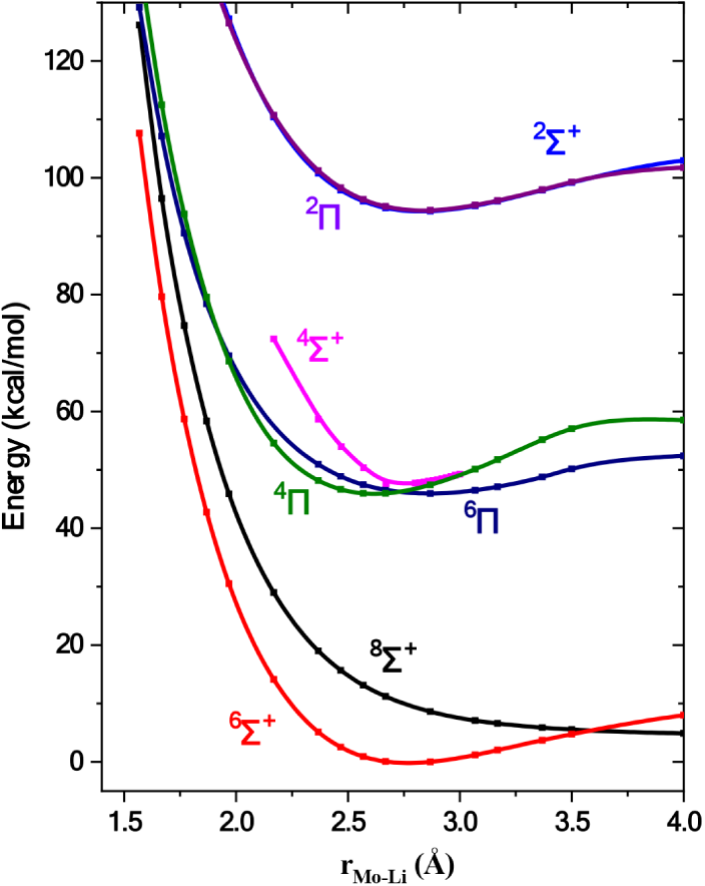
Potential energy curves of the low-lying states of MoLi at the CASSCF/aug-cc-pwCV5Z­(−PP) level. All energies are referenced to the ground state’s minimum.

For the ground state, the leading equilibrium CASSCF configuration is |XΣ+6⟩=0.93|1σ22σ11δ+11πx11πy11δ−1⟩ while the Mulliken atomic distributions (Mo/Li) at the C-MRCI level, are 5s^1.19^5p_z_^0.07^­5p_x_^0.03^­5p_y_^0.03^­4d_z^2^_^0.98^­4d_xz_^0.97^­4d_yz_^0.97^­4d_x^2^–y^2^_^0.99^­4d_xy_^0.99^/2s^0.59^­2p_z_^0.08^­2p_x_^0.03^­2p_y_^0.03^. The molecular orbitals’ composition is depicted in Table 3, while the bonding can be summarized in the following vbL diagram (Scheme 1) based on the leading CSF, population distributions, and molecular orbitals’ composition. More specifically, one σ bond is formed between the 5s electron of Mo and the 2s electron of Li. The results obtained from our calculations are presented in Table 2. Based on our dissociation energies, we conclude that a weak covalent bond is formed. The calculated dissociation energy values range from 19.6 to 24.4 kcal/mol depending on the calculated method. Our best D_e_ is obtained at the C-RCCSD­(T)/aug-cc-pwCV5Z level and is equal to 24.4 kcal/mol while at the same level, the corresponding ground state dissociation energy (D_0_) is equal to 23.9 kcal/mol. Based on Mulliken population analysis at the C-MRCISD level, a total of about 0.25e^–^ are transferred from the Li atom to the Mo atom.

**Scheme 1:**
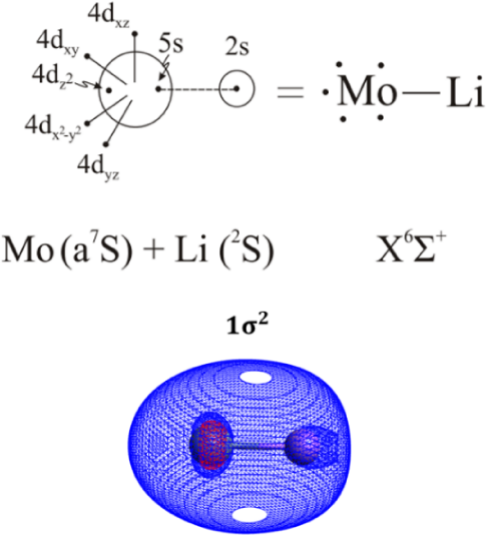
Bonding Scheme of the Ground State of MoLi

**Table 3:** Composition of the Molecular Orbitals (M.O.) of the Calculated States of MoX

M. O.	Atomic Orbitals	Atomic Orbitals
	**MoLi (X** ^**6**^ **Σ** ^**+**^ **)**: 0.93 $|{1\sigma }^{2}{2\sigma }^{1}{1\pi }_{x}^{1}1\pi_{y}^{1}{1\delta }_{+}^{1}{1\delta }_{-}^{1}$	**MoBe (X** ^**7**^ **Σ** ^**+**^ **)**: 0.95 $\left|1\sigma^{2}2\sigma^{1}3\sigma^{1}1\pi_{x}^{1}1\pi_{y}^{1}1\delta_{+}^{1}1\delta_{-}^{1}\right\rangle$
1σ	0.72 $\varphi_{2s\left(\mathrm{Li}\right)}$ + 0.66 $\varphi_{5s\left(\mathrm{Mo}\right)}$ – 0.32 $\varphi_{2p_{z}\left(\mathrm{Li}\right)}$	0.87 $\varphi_{2s\left(\mathrm{Be}\right)}$ + 0.28 $\varphi_{5s\left(\mathrm{Mo}\right)}$ + 0.23 $\varphi_{4d_{z^{2}}\left(\mathrm{Mo}\right)}$ – 0.15 $\varphi_{2p_{z}\left(\mathrm{Be}\right)}$
2σ	0.96 $\varphi_{4d_{z^{2}}\left(\mathrm{Mo}\right)}$ – 0.28 $\varphi_{5s\left(\mathrm{Mo}\right)}$ + 0.20 $\varphi_{2s\left(\mathrm{Li}\right)}$ – 0.20 $\varphi_{2p_{z}\left(\mathrm{Li}\right)}$	0.62 $\varphi_{4d_{z^{2}}\left(\mathrm{Mo}\right)}$ + 0.48 $\varphi_{5s\left(\mathrm{Mo}\right)}$ – 0.44 $\varphi_{2p_{z}\left(\mathrm{Be}\right)}$ – 0.39 $\varphi_{2s\left(\mathrm{Be}\right)}$
		–0.75 $\varphi_{5s\left(\mathrm{Mo}\right)}$ + 0.66 $\varphi_{4d_{z^{2}}\left(\mathrm{Mo}\right)}$
1π_*x*_	1.00 $\varphi_{4d_{\mathrm{xz}}\left(Μο\right)}$	0.96 $\varphi_{4d_{\mathrm{xz}}\left(Μο\right)}$ + 0.16 $\varphi_{2p_{x}\left(\mathrm{Be}\right)}$
1π_*y*_	1.00 $\varphi_{4d_{\mathrm{yz}}\left(Μο\right)}$	0.96 $\varphi_{4d_{\mathrm{yz}}\left(Μο\right)}$ + 0.16 $\varphi_{2p_{y}\left(\mathrm{Be}\right)}$
1δ_+_	1.00 $\varphi_{4d_{x^{2}-y^{2}}\left(\mathrm{Mo}\right)}$	1.00 $\varphi_{4d_{x^{2}-y^{2}}\left(\mathrm{Mo}\right)}$
1δ_–_	1.00 $\varphi_{4d_{\mathrm{xy}}\left(\mathrm{Mo}\right)}$	1.00 $\varphi_{4d_{\mathrm{xy}}\left(\mathrm{Mo}\right)}$
	**MoB (** *X* ^**6**^ **Π)**: $0.91/\sqrt{2}\left|1\sigma^{2}2\sigma^{1}3\sigma^{1}\left(1\pi_{x}^{2}1\pi_{y}^{1}+1\pi_{x}^{1}1\pi_{y}^{2}\right)1\delta_{+}^{1}1\delta_{-}^{1}\right\rangle$	**MoB (Α** ^**6**^ **Σ** ^**+**^ **)**: $0.92\left|1\sigma^{2}2\sigma^{2}1\delta_{+}^{1}3\sigma^{1}1\pi_{x}^{1}1\pi_{y}^{1}1\delta_{-}^{1}\right\rangle$
1σ	0.88 $\varphi_{2s\left(B\right)}$ + 0.31 $\varphi_{4d_{z^{2}}\left(\mathrm{Mo}\right)}$ + 0.21 $\varphi_{5s\left(\mathrm{Mo}\right)}$	0.85 $\varphi_{2s\left(B\right)}$ + 0.33 $\varphi_{4d_{z^{2}}\left(\mathrm{Mo}\right)}$ - 0.22 $\varphi_{2p_{z}\left(B\right)}$
2σ	–0.66 $\varphi_{2p_{z}\left(B\right)}$ + 0.58 $\varphi_{4d_{z^{2}}\left(\mathrm{Mo}\right)}$ – 0.35 $\varphi_{2s\left(B\right)}$ + 0.29 $\varphi_{5s\left(\mathrm{Mo}\right)}$	–0.64 $\varphi_{2p_{z}\left(B\right)}$ + 0.57 $\varphi_{4d_{z^{2}}\left(\mathrm{Mo}\right)}$ – 0.40 $\varphi_{2s\left(B\right)}$
3σ	0.82 $\varphi_{5s\left(\mathrm{Mo}\right)}$ – 0.51 $\varphi_{4d_{z^{2}}\left(\mathrm{Mo}\right)}$	0.87 $\varphi_{5s\left(\mathrm{Mo}\right)}$ – 0.49 $\varphi_{4d_{z^{2}}\left(\mathrm{Mo}\right)}$
1π_*x*_	0.75 $\varphi_{4d_{\mathrm{xz}}\left(Μο\right)}$ + 0.49 $\varphi_{2p_{x}\left(B\right)}$	0.86 $\varphi_{4d_{\mathrm{xz}}\left(Μο\right)}$ + 0.34 $\varphi_{2p_{x}\left(B\right)}$
1π_*y*_	0.75 $\varphi_{4d_{\mathrm{yz}}\left(Μο\right)}$ + 0.49 $\varphi_{2p_{y}\left(B\right)}$	0.86 $\varphi_{4d_{\mathrm{yz}}\left(Μο\right)}$ + 0.34 $\varphi_{2p_{y}\left(B\right)}$
1δ_+_	1.00 $\varphi_{4d_{x^{2}-y^{2}}\left(\mathrm{Mo}\right)}$	1.00 $\varphi_{4d_{x^{2}-y^{2}}\left(\mathrm{Mo}\right)}$
1δ_–_	1.00 $\varphi_{4d_{\mathrm{xy}}\left(\mathrm{Mo}\right)}$	1.00 $\varphi_{4d_{\mathrm{xy}}\left(\mathrm{Mo}\right)}$
	**MoC (X** ^**3**^ **Σ** ^**–**^ **)**: 0.91 $\left|1\sigma^{2}2\sigma^{2}1\delta_{+}^{1}1\pi_{x}^{2}1\pi_{y}^{2}1\delta_{-}^{1}\right\rangle$	**MoN (X** ^**4**^ **Σ** ^**–**^ **)**: 0.93 $\left|1\sigma^{2}2\sigma^{2}1\delta_{+}^{1}3\sigma^{1}1\pi_{x}^{2}1\pi_{y}^{2}1\delta_{-}^{1}\right\rangle$
1σ	0.88 $\varphi_{2s\left(C\right)}$ + 0.37 $\varphi_{4d_{z^{2}}\left(\mathrm{Mo}\right)}$ + 0.20 $\varphi_{5s\left(\mathrm{Mo}\right)}$ – 0.16 $\varphi_{2p_{z}\left(C\right)}$	0.89 $\varphi_{2s\left(N\right)}$ – 0.47 $\varphi_{4p_{z}\left(\mathrm{Mo}\right)}$
2σ	0.68 $\varphi_{4d_{z^{2}}\left(\mathrm{Mo}\right)}$ – 0.62 $\varphi_{2p_{z}\left(C\right)}$ – 0.22 $\varphi_{2s\left(C\right)}$	0.75 $\varphi_{2p_{z}\left(N\right)}$ – 0.62 $\varphi_{4d_{z^{2}}\left(\mathrm{Mo}\right)}$ – 0.18 $\varphi_{5s\left(\mathrm{Mo}\right)}$
3σ		0.94 $\varphi_{5s\left(\mathrm{Mo}\right)}$ – 0.27 $\varphi_{4d_{z^{2}}\left(\mathrm{Mo}\right)}$ – 0.21 $\varphi_{5p_{z}\left(Μο\right)}$
1π_*x*_	0.64 $\varphi_{4d_{\mathrm{xz}}\left(Μο\right)}$ + 0.59 $\varphi_{2p_{x}\left(C\right)}$	0.69 $\varphi_{2p_{x}\left(N\right)}$ + 0.53 $\varphi_{4d_{\mathrm{xz}}\left(Μο\right)}$
1π_*y*_	0.64 $\varphi_{4d_{\mathrm{yz}}\left(Μο\right)}$ + 0.59 $\varphi_{2p_{y}\left(C\right)}$	0.69 $\varphi_{2p_{y}\left(N\right)}$ + 0.53 $\varphi_{4d_{\mathrm{yz}}\left(Μο\right)}$
1δ_+_	1.00 $\varphi_{4d_{x^{2}-y^{2}}\left(\mathrm{Mo}\right)}$	1.00 $\varphi_{4d_{x^{2}-y^{2}}\left(\mathrm{Mo}\right)}$
1δ_–_	1.00 $\varphi_{4d_{\mathrm{xy}}\left(\mathrm{Mo}\right)}$	1.00 $\varphi_{4d_{\mathrm{xy}}\left(\mathrm{Mo}\right)}$
	**MoO (X** ^**5**^ **Π)**: $0.94/\sqrt{2}\left|1\sigma^{2}2\sigma^{2}3\sigma^{1}1\pi_{x}^{2}1\pi_{y}^{2}\left(2\pi_{x}^{1}+2\pi_{y}^{1}\right)1\delta_{+}^{1}1\delta_{-}^{1}\right\rangle$	**MoO (** ^**5**^ **Σ** ^**–**^ **)**: $0.95\left|1\sigma^{2}2\sigma^{2}3\sigma^{1}4\sigma^{1}1\pi_{x}^{2}1\pi_{y}^{2}1\delta_{+}^{1}1\delta_{-}^{1}\right\rangle$
1σ	0.94 $\varphi_{2s\left(0\right)}$	0.93 $\varphi_{2s\left(O\right)}$
2σ	0.82 $\varphi_{2p_{z}\left(O\right)}$ – 0.47 $\varphi_{4d_{z^{2}}\left(\mathrm{Mo}\right)}$ + 0.20 $\varphi_{4p_{z}\left(\mathrm{Mo}\right)}$ – 0.12 $\varphi_{5s\left(\mathrm{Mo}\right)}$	0.86 $\varphi_{2p_{z}\left(O\right)}$ – 0.40 $\varphi_{4d_{z^{2}}\left(\mathrm{Mo}\right)}$
3σ	0.91 $\varphi_{5s\left(\mathrm{Mo}\right)}$ – 0.34 $\varphi_{4d_{z^{2}}\left(\mathrm{Mo}\right)}$ – 0.17 $\varphi_{5p_{z}\left(\mathrm{Mo}\right)}$	–0.83 $\varphi_{4d_{z^{2}}\left(\mathrm{Mo}\right)}$ – 0.38 $\varphi_{2p_{z}\left(O\right)}$ + 0.22 $\varphi_{5p_{z}\left(\mathrm{Mo}\right)}$
4σ		–0.89 $\varphi_{5s\left(\mathrm{Mo}\right)}$ + 0.39 $\varphi_{5p_{z}\left(\mathrm{Mo}\right)}$
1π_*x*_	0.78 $\varphi_{2p_{x}\left(O\right)}$ + 0.39 $\varphi_{4d_{\mathrm{xz}}\left(Μο\right)}$	0.78 $\varphi_{2p_{x}\left(O\right)}$ + 0.40 $\varphi_{4d_{\mathrm{xz}}\left(Μο\right)}$
2π_*x*_	{0.93 $\varphi_{4d_{\mathrm{yz}}\left(Μο\right)}$ – 0.53 $\varphi_{2p_{y}\left(O\right)}$ }	
1π_*y*_	0.78 $\varphi_{2p_{y}\left(O\right)}$ + 0.39 $\varphi_{4d_{\mathrm{yz}}\left(Μο\right)}$	0.78 $\varphi_{2p_{y}\left(O\right)}$ + 0.40 $\varphi_{4d_{\mathrm{yz}}\left(Μο\right)}$
2π_*y*_	0.93 $\varphi_{4d_{\mathrm{yz}}\left(Μο\right)}$ – 0.53 $\varphi_{2p_{y}\left(O\right)}$	
1δ_+_	1.00 $\varphi_{4d_{x^{2}-y^{2}}\left(\mathrm{Mo}\right)}$	1.00 $\varphi_{4d_{x^{2}-y^{2}}\left(\mathrm{Mo}\right)}$
1δ_–_	1.00 $\varphi_{4d_{\mathrm{xy}}\left(\mathrm{Mo}\right)}$	1.00 $\varphi_{4d_{\mathrm{xy}}\left(\mathrm{Mo}\right)}$
	**MoF (X** ^**6**^ **Σ** ^**+**^ **)**: $0.99\left|1\sigma^{2}2\sigma^{2}3\sigma^{1}1\pi_{x}^{2}2\pi_{x}^{1}1\pi_{y}^{2}2\pi_{y}^{1}1\delta_{+}^{1}1\delta_{-}^{1}\right\rangle$	**MoF (** ^**6**^ **Π)**: $0.99/\sqrt{2}\left|1\sigma^{2}2\sigma^{2}3\sigma^{1}4\sigma^{1}1\pi_{x}^{2}1\pi_{y}^{2}\left(2\pi_{x}^{1}+2\pi_{y}^{1}\right)1\delta_{+}^{1}1\delta_{-}^{1}\right\rangle$
1σ	0.96 $\varphi_{2s\left(F\right)}$ – 0.19 $\varphi_{2p_{z}\left(F\right)}$	1.00 $\varphi_{2s\left(F\right)}$
2σ	0.86 $\varphi_{2p_{z}\left(F\right)}$ + 0.24 $\varphi_{4p_{z}\left(\mathrm{Mo}\right)}$ – 0.24 $\varphi_{4d_{z^{2}}\left(\mathrm{Mo}\right)}$	0.81 $\varphi_{2p_{z}\left(F\right)}$ – 0.34 $\varphi_{4s\left(\mathrm{Mo}\right)}$ – 0.15 $\varphi_{4d_{z^{2}}\left(\mathrm{Mo}\right)}$
3σ	0.90 $\varphi_{5s\left(\mathrm{Mo}\right)}$ – 0.43 $\varphi_{4d_{z^{2}}\left(\mathrm{Mo}\right)}$	1.00 $\varphi_{4d_{z^{2}}\left(\mathrm{Mo}\right)}$
4σ		1.00 $\varphi_{5s\left(\mathrm{Mo}\right)}$
1π_*x*_	1.00 $\varphi_{2p_{x}\left(F\right)}$	0.90 $\varphi_{2p_{x}\left(F\right)}$ + 0.11 $\varphi_{4d_{\mathrm{xz}}\left(Μο\right)}$
2π_*x*_	1.00 $\varphi_{4d_{\mathrm{xz}}\left(Μο\right)}$	1.00 $\varphi_{4d_{\mathrm{xz}}\left(Μο\right)}$
1π_*y*_	1.00 $\varphi_{2p_{y}\left(F\right)}$	0.90 $\varphi_{2p_{y}\left(F\right)}$ + 0.11 $\varphi_{4d_{\mathrm{yz}}\left(Μο\right)}$
2π_*y*_	1.00 $\varphi_{4d_{\mathrm{yz}}\left(Μο\right)}$	{1.00 $\varphi_{4d_{\mathrm{xz}}\left(Μο\right)}$ }
1δ_+_	1.00 $\varphi_{4d_{x^{2}-y^{2}}\left(\mathrm{Mo}\right)}$	1.00 $\varphi_{4d_{x^{2}-y^{2}}\left(\mathrm{Mo}\right)}$
1δ_–_	1.00 $\varphi_{4d_{\mathrm{xy}}\left(\mathrm{Mo}\right)}$	1.00 $\varphi_{4d_{\mathrm{xy}}\left(\mathrm{Mo}\right)}$

Regarding the dipole moment, there is a significant difference between the expectation values and the values obtained via the finite field method at the MRCISD and C-MRCISD levels. In particular, the finite field dipole moment ranges from 3.30 to 3.74 D, while the expectation value ranges from 2.08 to 2.69 D. It should be noted, however, that the finite field method provides in general more accurate results.^61^ Note that although the definitions of dipole moment as an expectation value or as a value calculated via finite field method are equivalent to the limit, in most other cases they differ. This is because < μ> is a functional of the wave function, while in μ_FF_ the wave function is indirectly involved through the energy E. However, for the CASSCF method, which can be regarded as a full CI within the active space, the < μ> and μ_FF_ are exactly the same values, as it is expected. Finally, comparing our three DFT methods with the C-MRCISD+Q and C-RCCSD­(T) ones, it turns out that the B3LYP functional shows the best agreement for r_e_, D_e_, and *ω*_e_, while MN15 shows the best agreement for μ.

### 3.2 MoBe

The interaction between the ground state fragments, Mo (a^7^S) + Be (^1^S), results in the formation of one electronic state of ^7^Σ^+^ symmetry. Given that there are no previous studies on this molecule, nine low-lying states, i.e., ^1, 2, 3, 5, 7^Σ^+^, ^1, 2, 3, 5, 7^Π and ^1^Φ, were calculated at the CASSCF level, see Figure 3. Despite being repulsive at the CASSCF level, further investigation at the C-MRCI level (see Figure 1) revealed that the state of ^7^Π^+^ symmetry is bound. Since it is well separated from the rest of the states, it represents the ground state of this molecule. The leading equilibrium CASSCF configuration, followed by the Mulliken atomic distributions (Mo/Be) at the C-MRCI level of theory, are |XΣ+7⟩=0.95|1σ21δ+12σ13σ11πx11πy11δ−1⟩ and 5s^0.96^5p_z_^0.13^­5p_x_^0.01^­5p_y_^0.01^­4d_z^2^_^0.99^­4d_xz_^0.95^­4d_yz_^0.95^­4d_x^2^–y^2^_^0.99^­4d_xy_^0.99^/2s^1.47^­2p_z_^0.33^ ­2p_x_^0.08^2p_y_^0.08^. The molecular orbitals’ composition is depicted in Table 3. Scheme 2 provides a pictorial representation of the bonding scheme of this state, based on the leading CSF, atomic Mulliken charge distributions, and the composition of molecular orbitals. To be more specific, a dative σ-bond is formed between the singly occupied 4d_z^2^_ orbital of Mo and the empty 2p_z_ orbital of Be, i.e., 2σ^1^ bond, see Scheme 2.

**Scheme 2:**
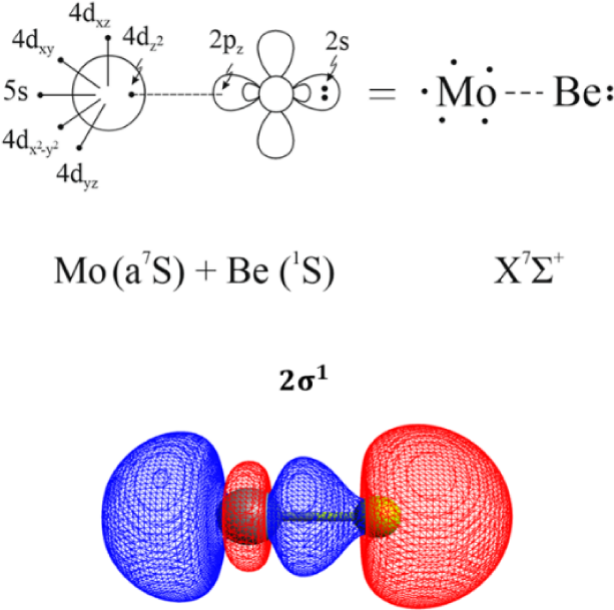
Bonding Scheme of the Ground State of MoBe

**Figure 3:**
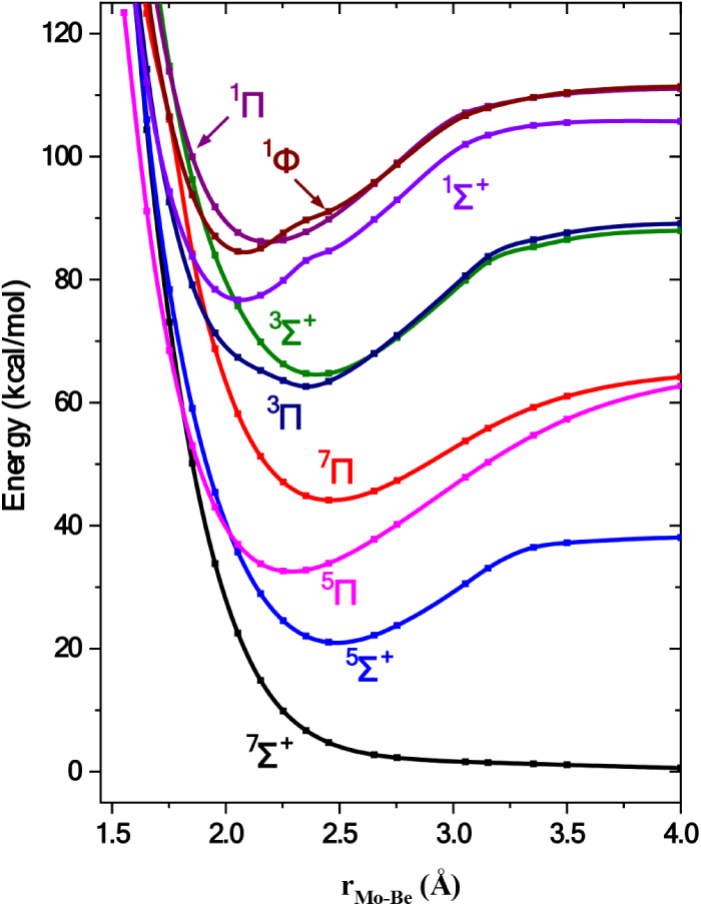
Potential energy curves of the low-lying states of MoBe at the CASSCF/aug-cc-pwCV5Z­(−PP) level. All energies are referenced to the dissociation limit of the ground state fragments, Mo (a^7^S) + Be (^1^S).

Table 2 summarizes the results obtained from our calculations. Our best methodology, C-RCCSD­(T), predicts a binding energy (D_e_) with respect to the adiabatic channel, Mo (a^7^S) + Be (^1^S), of 14.4 kcal/mol at r_e_ = 2.452 Å. Thus, a weak single-electron dative bond is formed. It should be noted that all three DFT methodologies provide significantly overestimated dissociation energies ranging from 19.2 to 24.4 kcal/mol. Regarding bond distance, the TPSSh functional shows the best agreement with the C-RCCSD­(T) method.

### 3.3 MoB

In the case of MoB, the ground state fragments Mo (a^7^S) + B (^2^P), give rise to ^6^Π and ^6^Σ^+^ electronic states, see Figure 4. The ground state, *X* ^6^Π, lies 5.38 kcal/mol below the *A* ^6^Σ^+^ state, see Table 2. Their leading equilibrium CASSCF configurations are |*X* ^6^Π⟩ = $\frac{0.91}{\sqrt{2}}\left|1\sigma^{2}1\delta_{+}^{1}2\sigma^{1}3\sigma^{1}\left(1\pi_{x}^{2}1\pi_{y}^{1}+\left(1\pi_{x}^{1}1\pi_{y}^{2}\right)\right)1\delta_{-}^{1}\right\rangle$ and |*A* ^6^Σ^+^ = $0.92|1\sigma^{2}2\sigma^{2}1\delta_{+}^{1}3\sigma^{1}1\pi_{x}^{1}1\pi_{y}^{1}1\delta_{-}^{1}\rangle$ , while their atomic distributions at the C-MRCI level of theory are *X* ^6^Π: 5s^0.89^5p_z_^0.13^­5p_x_^0.03^­5p_y_^0.01^­4d_z^2^_^0.91^­4d_xz_^1.20^­4d_yz_^0.75^­4d_x^2^–y^2^_^0.99^­4d_xy_^0.99^/2s^1.49^­2p_z_^0.53^­2p_x_^0.75^2p_y_^0.26^ and *A* ^6^Σ^+^: 5s^0.89^­5p_z_^0.13^­5p_x_^0.01^­5p_y_^0.01^­4d_z^2^_^1.17^­4d_xz_^0.80^­4d_yz_^0.80^­4d_x^2^–y^2^_^0.99^4d_xy_^0.99^/2s^1.77^­2p_z_^0.92^­2p_x_^0.21^­2p_y_^0.21^. The valence molecular orbitals (Table 3) of these states show that the bonding of both states consists of two bonds, see Scheme 3. In particular, the bonding in the *X* ^6^Π state comprises one two-electron π bond, one one-electron σ bond, and one one-electron π bond, while a total of about 0.11e^–^ are transferred from the metal to the B atom. The bonding in the A^6^Σ+ state comprises one two-electron σ bond, and two one-electron π bonds, whereas a total of about 0.19e^–^ are transferred from the metal to the B atom.

**Scheme 3:**
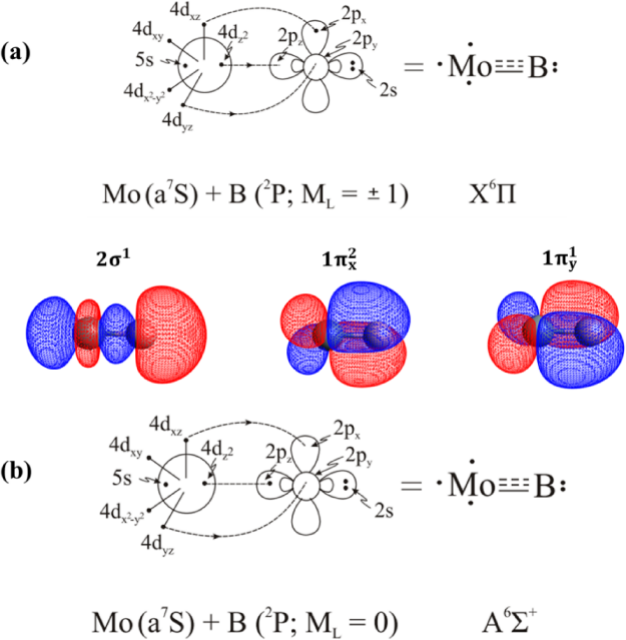
Bonding Schemes of the *X* ^6^Π (a) and *A* ^6^Σ^+^ (b) States of MoB

**Figure 4:**
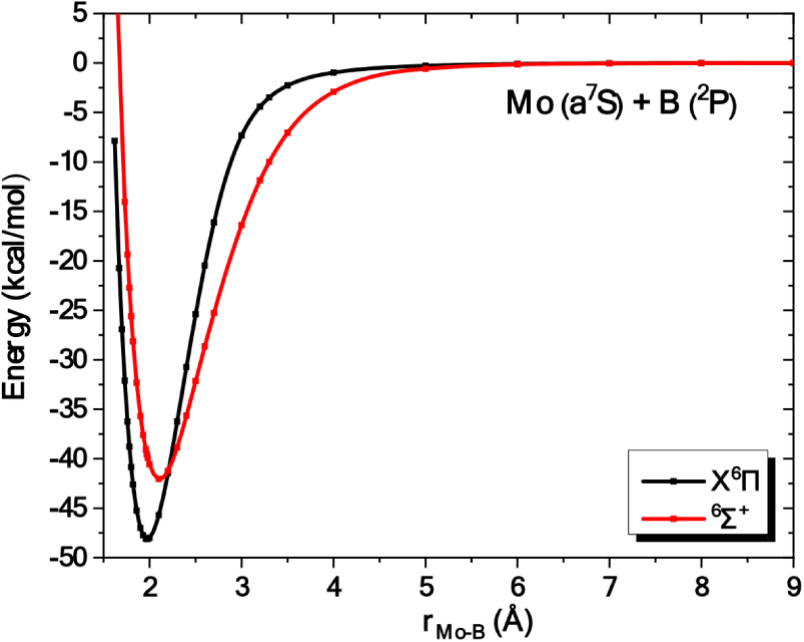
Potential energy curves of the *X* ^6^Π and ^6^Σ^+^ states of MoB at the C-MRCISD+Q/aug-cc-pwCV5Z­(−PP) level. All energies are referenced to the dissociation limit of the ground state fragments, Mo (a^7^S) + B (^2^P).

The C-RCCSD­(T) dissociation energies of *X* ^6^Π and *A* ^6^Σ^+^ states are D_e_ = 47.2and41.8kcal/mol at r_e_ = 1.959and2.104Å, respectively, while the C-MRCISD+Q D_e_ values are 48.2 and 42.1 kcal/mol, see Table 2. These values are in very good agreement with the CASPT2/Q-ζ ANO-RCC results of Borin and Gobbo.^12^ The PECs of these two states are plotted in Figure 4; they correlate to the ground state products and retain this character along the interatomic distance r_Mo‑B_. Finally, the dipole moments of both states are quite similar as they are equal to 2.47[2.31] D and 2.80[2.24] D at the C-MRCISD+Q­[C-RCCSD­(T)] levels.

### 3.4 MoC

The ground state of MoC is well separated from the rest of the electronic states.^15,19,23^ It is of ^3^Σ^–^ symmetry and correlates to Mo (a^5^S) + C (^3^P), see Figure 1. The leading equilibrium CASSCF configuration is |XΣ−3⟩=0.91|1σ22σ21δ+11πx21πy21δ−1⟩ and the Mulliken atomic distributions (Mo/C) at C-MRCI are 5s^0.20^5p_z_^0.09^­5p_x_^0.02^­5p_y_^0.02^­4d_z^2^_^1.26^­4d_xz_^1.06^­4d_yz_^1.06^­4d_x^2^–y^2^_^0.99^­4d_xy_^0.99^/2s^1.75^­2p_z_^0.71^­2p_x_^0.90^2p_y_^0.90^. The composition of the molecular orbitals is depicted in Table 3.

As we can see from the atomic populations above, there is a strong Mulliken charge transfer of 0.75e^–^ from the 5s orbital of Mo to the 2P_z_ orbital of C, indicating that equilibrium is dominated by an ionic picture, i.e., Mo^+^ + C^–^. The bonding in this state can be captured by the following vbL diagram (Scheme 4) based on the leading CSF, the atomic Mulliken distributions, and the molecular orbitals’ composition. As mentioned in our recent work,^23^ both pictures (covalent and ionic) have been considered. It should be noted, however, that the large dipole moment values obtained from our calculations imply that the ionic picture is dominant, see Table 2.

**Scheme 4:**
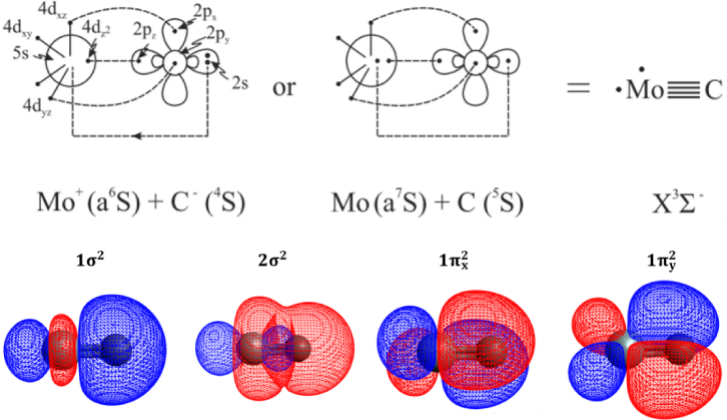
Bonding Scheme of the Ground State of MoC

The bonding in the *X* ^3^Σ^–^ state comprises two two-electron σ and two two-electron π bonds, thereby giving rise to a full quadruple bond. That is quite interesting as quadruple bonds are rare for main group elements. It is worth mentioning that the electronic states of Α^3^Δ, *a* ^3^Γ, *c* ^1^Δ, and *d* ^1^Σ^+^ symmetries also possess a full quadruple bond (σ^2^σ^2^π^2^π^2^) character.^23^ The dissociation energy (D_e_) with respect to adiabatic and ground state products is equal to 149.2 and 115.0 kcal/mol, respectively. By considering the complete basis set (CBS) limit, these values become equal to 152.5 and 118.3 kcal/mol. That is, the contribution of CBS extrapolation to dissociation energy equals 3.3 kcal/mol. Our extrapolated ground state dissociation energy is in excellent agreement with the corresponding experimental measurement of 118.4 ± 0.07 kcal/mol.^23^

### 3.5 MoN

The lowest state of MoN is of ^4^Σ^–^ symmetry.^24–29^ The *X* ^4^Σ^–^ state correlates to the ground state fragments Mo (a^7^S) + N (^4^S) and retains this configuration along the interatomic distance $r_{\mathrm{Mo‐N}}$ . The leading equilibrium CASSCF configuration is |XΣ−4⟩=0.93|1σ22σ21δ+13σ11πx21πy21δ−1⟩ and the C-MRCI atomic Mulliken populations (Mo/N) are 5s^0.20^­5p_z_^0.09^­5p_x_^0.02^­5p_y_^0.02^­4d_z^2^_^1.26^­4d_xz_^1.06^­4d_yz_^1.06^­4d_x^2^–y^2^_^0.99^­4d_xy_^0.99^/2s^1.75^­2p_z_^0.71^­2p_x_^0.90^2p_y_^0.90^. The composition of the molecular orbitals is depicted in Table 3 showing that the bonding consists of one two-electron σ and two two-electron π bonds, thereby giving rise to a full triple bond, see Scheme 5. Also, a total of about 0.49e^–^ are transferred from the metal to the N atom according to the total Mulliken populations. Our results are gathered in Table 2.

**Scheme 5:**
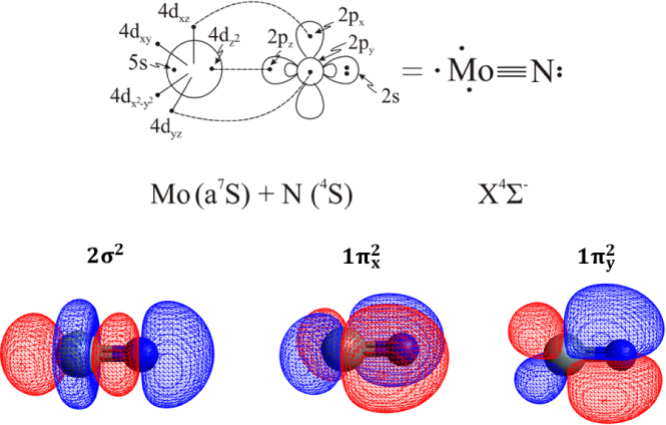
Bonding Scheme of the Ground State of MoN

The dissociation energy of the *X* state has been found equal to 123.7[122.6] kcal/mol at the C-MRCISD+Q­[C-RCCSD­(T)] levels. At the same levels, r_e_ **=** 1.635[1.627] Å, while *μ*_FF_ = 3.11[3.09] D. For the DFT methods employed in this work, it turns out that B3LYP and TPSSh functionals are in excellent agreement with our C-MRCISD+Q and C-RCCSD (T) results, while MN15 overestimates dissociation energies by 10 kcal/mol and underestimates equilibrium distances by 0.02 Å, see Table 2.

Finally, comparing the ground state of MoN with the ground states of FeC (X^3^ **Δ**) and FeC^+^ (X^2^ **Δ**)) that also exhibit a triple-bond character,^62^ MoN is more bound as D_e_(MoN) = 123.7cal/mol while D_e_(FeC) = 90.5kcal/mol and D_e_(FeC^+^) = 109.0kcal/mol. Thus, the ability of Mo atom to form strong bonds is highlighted in this case.

### 3.6 MoO

MoO has been studied both theoretically and experimentally.^24,28,30–37,41,42^ On the theoretical side, the calculated dissociation energies (D_e_) range from 84.6 to 151.0 kcal/mol while on the experimental side, D_0_ has been found equal to 125.9 ± 0.4,^31^ 124.85 ^32^ and 125.4 kcal/mol,^41^ see Table 1. In this study, we aim to provide a benchmark value for the dissociation energy of this system.

The ground state, *X* ^5^Π, as well as the lowest excited state (of ^5^Σ^–^ symmetry) correlated to the ground state products, Mo (a^7^S) + O (^3^P), have been calculated, see Figure 5 and Table 2. The CASSCF leading equilibrium configurations are |XΠ5⟩=0.94/2|1σ22σ23σ11πx21πy2(2πx1+2πy1)1δ+11δ−1⟩ and |Σ−5=0.95|1σ21δ+12σ13σ11πx21πy21δ−1⟩ . The Mulliken atomic distributions are *X* ^5^Π: 5s^0.81^5p_z_^0.07^­5p_x_^0.01^­5p_y_^0.04^­4d_z^2^_^0.69^­4d_xz_^0.59^­4d_yz_^1.13^­4d_x^2^–y^2^_^0.99^­4d_xy_^0.99^/2s^1.98^­2p_z_^1.44^­2p_x_^1.38^2p_y_^1.78^ and ^5^Σ^–^: 5s^0.82^­5p_z_^0.02^­5p_x_^0.01^­5p_y_^0.01^­4d_z^2^_^1.08^­4d_xz_^0.51^­4d_yz_^0.51^­4d_x^2^–y^2^_^0.99^­4d_xy_^0.99^/2s^1.97^­2p_z_^1.68^­2p_x_^1.44^2p_y_^1.44^. The composition of molecular orbitals for the states in question is given in Table 3. For the ground state, 0.3e^–^ are transferred from Mo to O via the σ-frame while 0.4e^–^ are moving from Mo to O through the π-frame according to the atomic populations at the C-MRCI level. In the excited state of ^5^Σ^–^ symmetry, 0.9e^–^ are transferred from Mo to O via the π-frame. The binding interaction in both states is captured by the following vbL diagrams (Scheme 6) based on the leading CSFs, population analysis, and the composition of molecular orbitals. Both states possess a double bond character (X^5^Π : σ^2^π^2^ ^5^Σ– : π^2^π^2^).

**Scheme 6:**
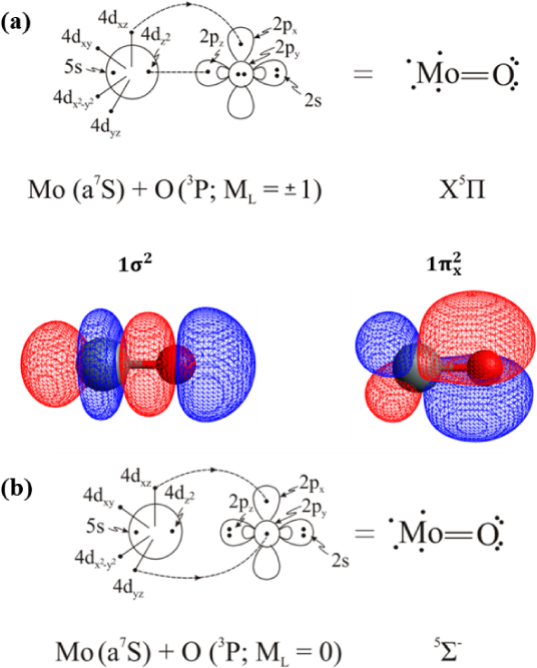
Bonding Schemes of the X^5^Π (a) and ^5^Σ^–^ (b) States of MoO

**Figure 5:**
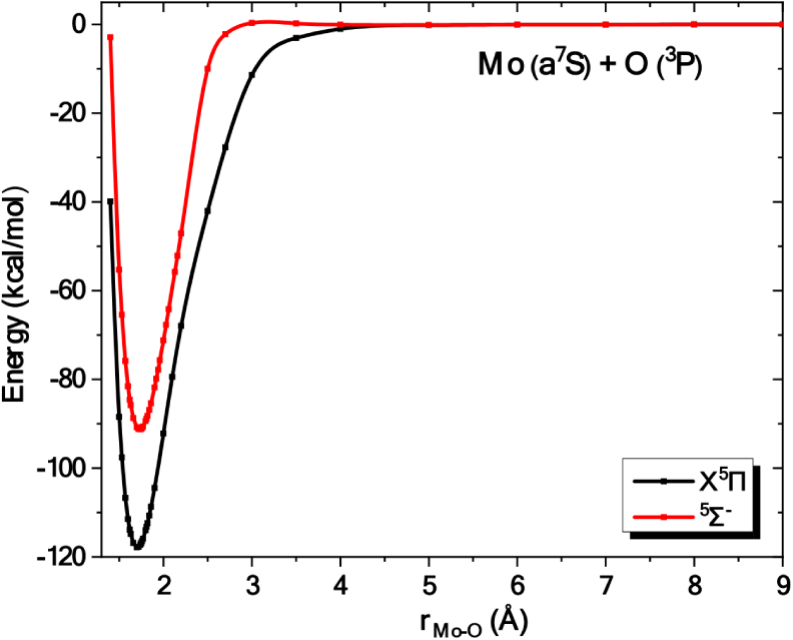
Potential energy curves of the *X* ^5^Π and ^5^Σ^–^ states of MoO at the C-MRCISD+Q/aug-cc-pwCV5Z­(−PP) level. All energies are referenced to the dissociation limit of the ground state fragments, Mo (a^7^S) + O (^3^P).

While all previous theoretical studies suggest a bond distance that ranges from 1.707 Å to 1.750 Å, our results are in reasonable agreement with the experimental value of 1.7129 Å for r_0_.^34^ As mentioned above, there is a significant discrepancy between the calculated dissociation energies reported in the literature and the experimentally determined ones. In particular, D_e_ ranges from 84.6 to 151.0 kcal/mol while the experimental value of D_0_ is approximately equal to 125 kcal/mol.^31,32,41^ The largest discrepancies are observed for the DFT data mainly due to the small basis sets being employed. Our DFT results predict equilibrium distances ranging from 1.678 to 1.699 Å and dissociation energies ranging from 123.5 to 131.5 kcal/mol, which are in good agreement with the corresponding experimental values.

Our best methodology, C-RCCSD­(T)/aug-cc-pV5Z­(−PP), yields a D_e_ (D_0_) value of 122.9 (121.5) kcal/mol, which is in very good agreement with the experimentally measured D_0_ values of 125.9 ± 0.4,^31^ 124.85 ^32^ and 125.4 kcal/mol.^41^ To provide a benchmark value for r_e_ and D_0_, the complete basis set (CBS) limit has been considered at the C-RCCSD­(T) level. More specifically, the CBS limit has been obtained using a sequence of weighted core–valence correlation consistent basis sets, i.e., aug-cc-pwCV*n*Z­(−PP), *n* = D, T, Q, and 5. The extrapolation Scheme 1s based on the following exponential formula^63,64^

$$f\left(n\right)=C_{0}+C_{1}e^{-C_{2}n},\lim_{n\to \infty }f\left(n\right)=C_{0}$$

The results are gathered in Table 4. The extrapolated D_0_ and r_e_ values (121.8 ± 0.1 kcal/mol and 1.6955 Å, respectively) are in pretty good agreement with the corresponding experimental values of 124.85 kcal/mol^32^ and 1.7129 Å.^34^ For the equilibrium distance, the percent deviation between theory and experiment is equal to 1%, whereas the corresponding percent deviation for D_0_ equals 2%. Note that the CBS limit is equivalent to the results obtained using the aug-cc-pwCV5Z basis set. Finally, the C-MRCISD+Q method provides an elongated (compared to CBS limit) r_e_ value of 1.706 Å, thereby reducing the deviation between theory and experiment to 0.4%.

**Table 4:** Bond Distances r_e_ (Å) and Adiabatic Dissociation Energies D_0_ (kcal/mol) of the Ground State, *X* ^5^Π, of MoΟ at the c-Rccsd­(t)/aug-Cc-pwCV*n*Z­(−PP), *N* = D(2), T(3), Q(4), and 5 Levels of Theory^a^ ^b^

	D(2)	T(3)	Q(4)	5	CBS
D_0_	110.6	118.4	120.7	121.5	**121.8 ± 0.1**
r_e_	1.715	1.701	1.697	1.696	**1.6955**
**Expt.** D_0_	124.85^a^				
**Expt.** r_e_	1.7129^b^				

a R2PI spectroscopy; ref. 32

b LIF and SVL spectroscopy; ref. 34

Comparing the electronic states of MoO studied in the present work with the corresponding electronic states of the isovalent MoS,^65^ the equilibrium distances of MoO (X^5^II : 1.706Å ^5^Σ^–^ : 1.732Å) are smaller than the ones of MoS (X^5^Π : 2.131Å ^5^Σ^–^: 2.145 Å) at the C-MRCISD+Q level. It should be noted at this point that both states of MoS exhibit a double-bond character. At the C-RCCSD­(T) level, the D_0_ values of MoO (X^5^Π : 121.8 ± 0.1kcal/mol ^5^Σ^–^ : 91.4 kcal/mol) are significantly larger than the ones of MoS (X^5^Π :90.8kcal/mol ^5^Σ^–^: 66.6 kcal/mol).

### 3.7 MoF

Two electronic states of *X* ^6^Σ^+^ and ^6^Π symmetry have been considered. Both of them correlate to the ground state atoms Mo (a^7^S) + F (^2^P). At equilibrium, the leading CASSCF configurations are |*X* ^6^Σ^+^ = $0.99|1\sigma^{2}2\sigma^{2}3\sigma^{2}4\sigma^{2}5\sigma^{2}1\delta_{+}^{1}6\sigma^{1}1\pi_{x}^{2}2\pi_{x}^{2}3\pi_{x}^{1}1\pi_{y}^{2}2\pi_{y}^{2}3\pi_{y}^{1}1\delta_{-}^{1}\rangle$ and ${|}^{6}\Pi \rangle =0.99/\sqrt{2}|1\sigma^{2}2\sigma^{2}$ $3\sigma^{1}4\sigma^{1}$ $1\pi_{x}^{2}1\pi_{y}^{2}\left(2\pi_{x}^{1}+2\pi_{y}^{1}\right)1\delta_{+}^{1}1\delta_{-}^{1}\rangle$ . The atomic distributions (Mo/F) at the C-MRCI level are 5s^0.76^5p_z_^0.03^­5p_x_^0.04^­5p_y_^0.04^­4d_z^2^_^0.40^­4d_xz_^1.01^­4d_yz_^1.01^­4d_x^2^–y^2^_^1.00^­4d_xy_^1.00^/ 2s^2.02^­2p_z_^1.77^­2p_x_^1.92^2p_y_^1.92^ and 5s^0.84^5p_z_^0.25^­5p_x_^0.02^­5p_y_^0.02^­4d_z^2^_^0.98^­4d_xz_^1.02^­4d_yz_^0.10^­4d_x^2^–y^2^_^0.99^­4d_xy_^0.99^/2s^2.03^­2p_z_^1.87^­2p_x_^1.93^2p_y_^1.88^. The composition of molecular orbitals for the states in question is depicted in Table 3.

The bonding scheme can be captured by the following vbL diagrams of Scheme 7, based on the leading CSFs, atomic Mulliken distributions, and the composition of molecular orbitals. The population analysis suggests that, in both states, equilibrium is dominated by an ionic picture, i.e., M0^+^ + F^–^. More specifically, in the *X* ^6^Σ^+^ state, 0.6e^–^ migrate from the metal to the 2p_*z*_ orbital of F atom, whereas in the ^6^Π state, 0.8e^–^ are transferred from the metal to the 2p_*y*_ orbital of fluorine. Thus, the *in situ* M0^+^ cation finds itself in the first excited state, aD6. The bonding in the *X* ^6^Σ^+^ state comprises one dative two-electron σ bond plus one dative two-electron π bond in the ^6^Π state. As the interatomic distance is increased, the bond changes from ionic to covalent.

**Scheme 7:**
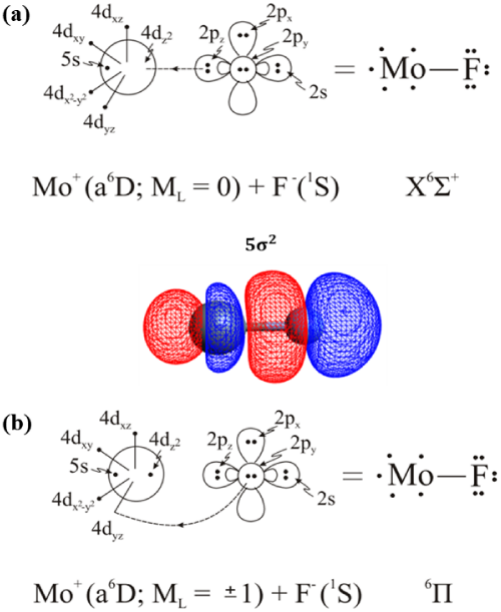
Bonding Schemes of the *X* ^6^Σ^+^ (a) and ^6^Π (b) States of MoF

For the *X* ^6^Σ^+^state, the molecule is dominated by an ionic picture at internuclear distances between 1.5 Å and 3.0 Å. Nevertheless, this state correlates to the ground state fragments, Mo (a^7^S) + F (^2^P), since it suffers from an avoided crossing near 3.5 Å. The charge of the 2p_*z*_ atomic orbital of F with respect to the internuclear distance, r_Mo–F_ is plotted in Figure 6a. A significant shift in the charge of the 2p_*z*_ atomic orbital of F takes place near 3.5 Å [ $Q_{2p_{z}\left(F\right)}$ = 1.82 → $Q_{2p_{z}\left(F\right)}$ = 1.06] denoting the transition from the ionic picture, Mo^+^ (a^6^D) + F^–^ (^1^S), to the covalent one, Mo (a^7^S) + F (^2^P), or in other words, the avoided crossing’s presence. This happens because the presence of two electrons in the 2p_*z*_ atomic orbital of F is indicative of the ionic picture [F^–^ (^1^S); $2p_{x}^{2}2p_{y}^{2}2p_{z}^{2}$ ], whereas the presence of one electron in the same orbital is indicative of the covalent picture [F (^2^P; M_L_ = 0); $2p_{x}^{2}2p_{y}^{2}2p_{z}^{1}$ ].

**Figure 6:**
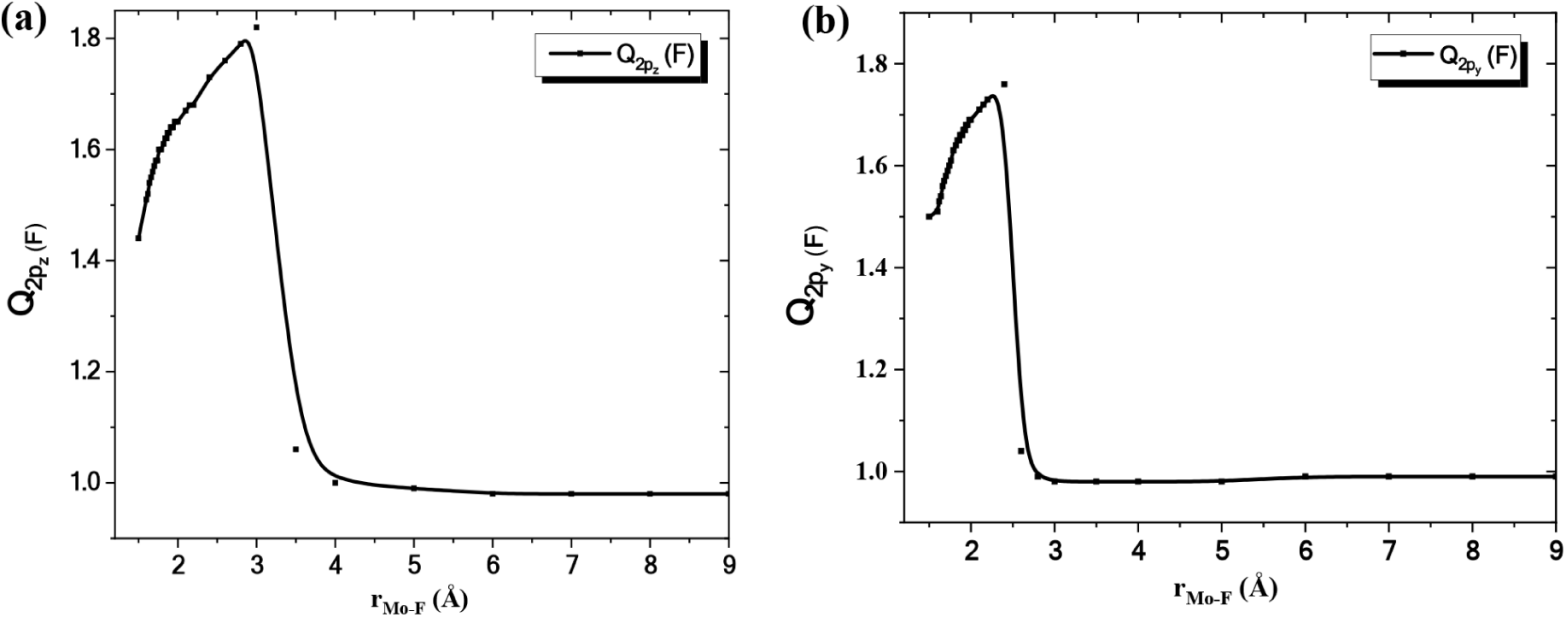
(a) The charge of the 2p_*z*_ orbital of F as a function of the internuclear distance, $r_{\mathrm{Mo}-F}$ (*X* ^6^Σ^+^ state); (b) The charge of the 2p_*y*_ orbital of F as a function of the internuclear distance, $r_{\mathrm{Mo}-F}$ (^6^Π state).

In the ^6^Π excited state, MoF is also dominated by an ionic picture at internuclear distances between 1.5 and 2.4 Å. However, the state in question also correlates to the ground state atoms, Mo (a^7^S) + F (^2^P), because of an avoided crossing near 2.6 Å, Figure 7. As evidence, we investigated the relationship between the charge of the 2p_*y*_ atomic orbital of F and the internuclear distance, r_Mo–F_, see Figure 6b. A significant shift in the charge of the 2p_*y*_ atomic orbital of F takes place near 2.6 Å [ $Q_{2p_{y}\left(F\right)}$ = 1.76 → $Q_{2p_{y}\left(F\right)}$ = 1.04] denoting the transition from the ionic picture, Mo^+^ (a^6^D) + F^–^F^–^ (^1^S), to the covalent one, Mo (a^7^S) + F (^2^P). The presence of two electrons in the 2p_*y*_ atomic orbital of F is indicative of the ionic picture [F^–^ (^1^S); $2p_{x}^{2}2p_{y}^{2}2p_{z}^{2}$ ], whereas the presence of one electron in the same orbital is indicative of the covalent picture [F (^2^P; M_L_ = +1); $2p_{x}^{2}2p_{y}^{1}2p_{z}^{2}$ ].

**Figure 7:**
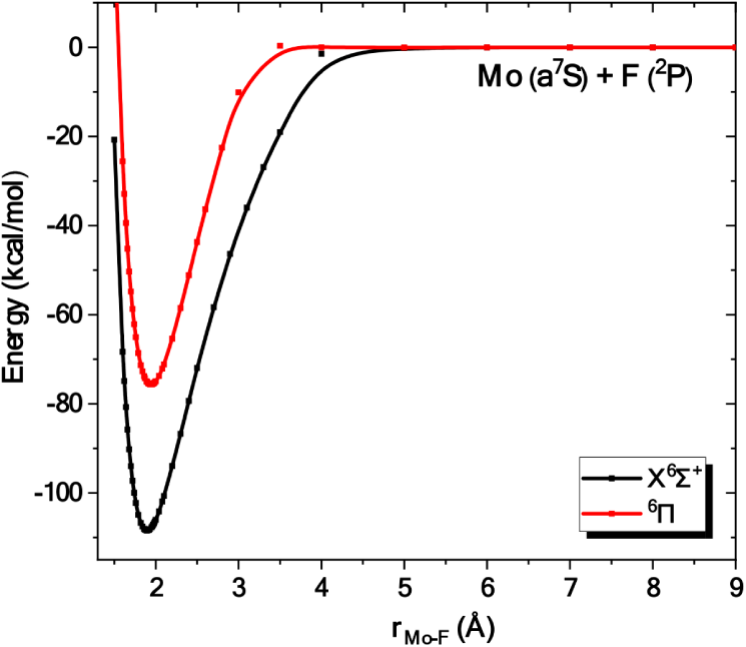
Potential energy curves of the *X* ^6^Σ^+^ and ^6^Π states of MoF at the C-MRCISD+Q/aug-cc-pwCV5Z­(−PP) level. All energies are referenced to the dissociation limit of the ground state fragments, Mo (a^7^S) + F (^2^P).

For both states, C-RCCSD­(T) dissociation energies are very large even though the bond is single. In particular, the corresponding values are D_e_ = 111.4 (*X* ^6^Σ^+^) and 79.2 (^6^Π) kcal/mol at r_e_ = 1.895­(X^6^Σ^+^)­and1.947­(^6^Π)­Å. These large dissociation energies of the single bond, which is predominantly ionic at equilibrium, can be explained by considering this bond as a charge-shift bonding come from a covalent-ionic resonance in MoF. Finally, we should emphasize the importance of taking the contribution of core–valence electrons [4s4p (Mo) + 1s (F)] into account in our calculations. More specifically, the right energetic ordering of the molecular orbitals would not have been possible at the CASSCF level if the electrons in question were not considered. Hence, C-MRCI calculations became feasible by including all 23 electrons [Mo (4s^2^4p^6^4d^5^5s^1^) + F (1s^2^2s^2^2p^5^)] in the active space.

### 3.8 TRENDS

Bond distances, dissociation energies, dipole moments as well as common spectroscopic constants of MoX species have been calculated via DFT, multireference configuration interaction and coupled-cluster methodologies. Figures 8 and 9 illustrate how these values change with respect to the X atoms.

**Figure 8:**
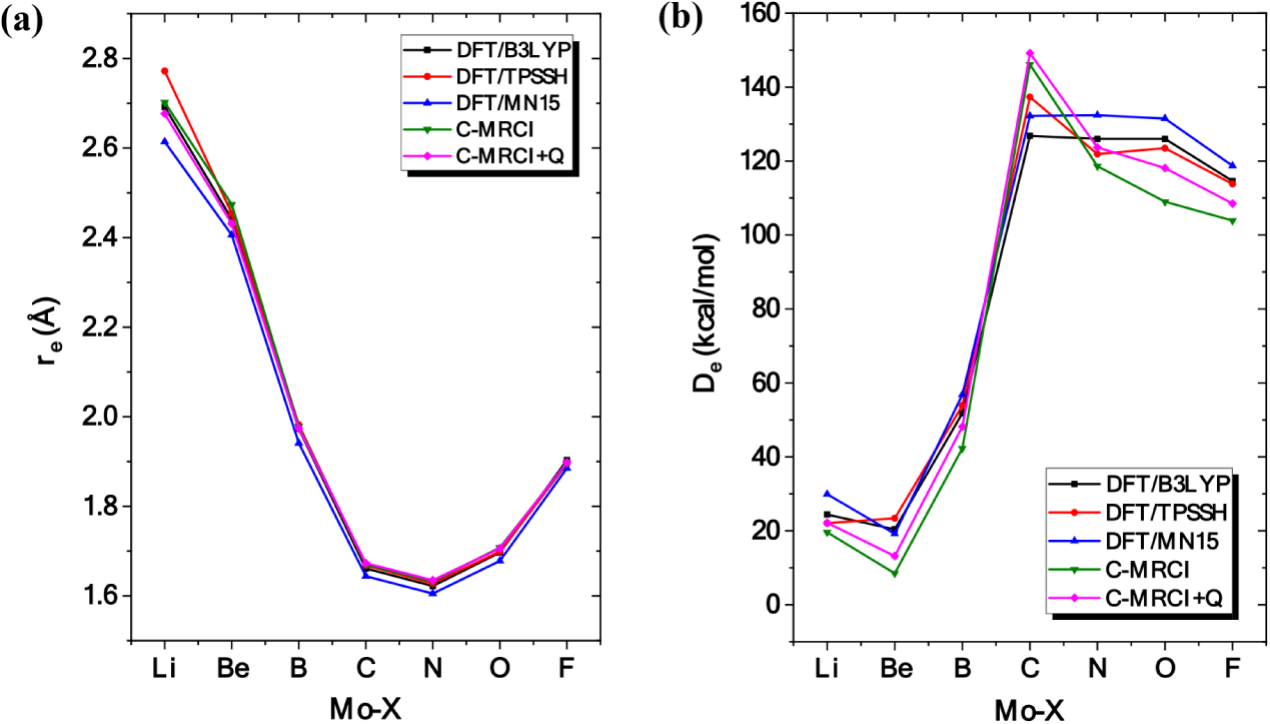
Bond multiplicity (a) and dissociation energy (b) of the ground states of MoX as a function of the X atom at different levels of theory.

**Figure 9:**
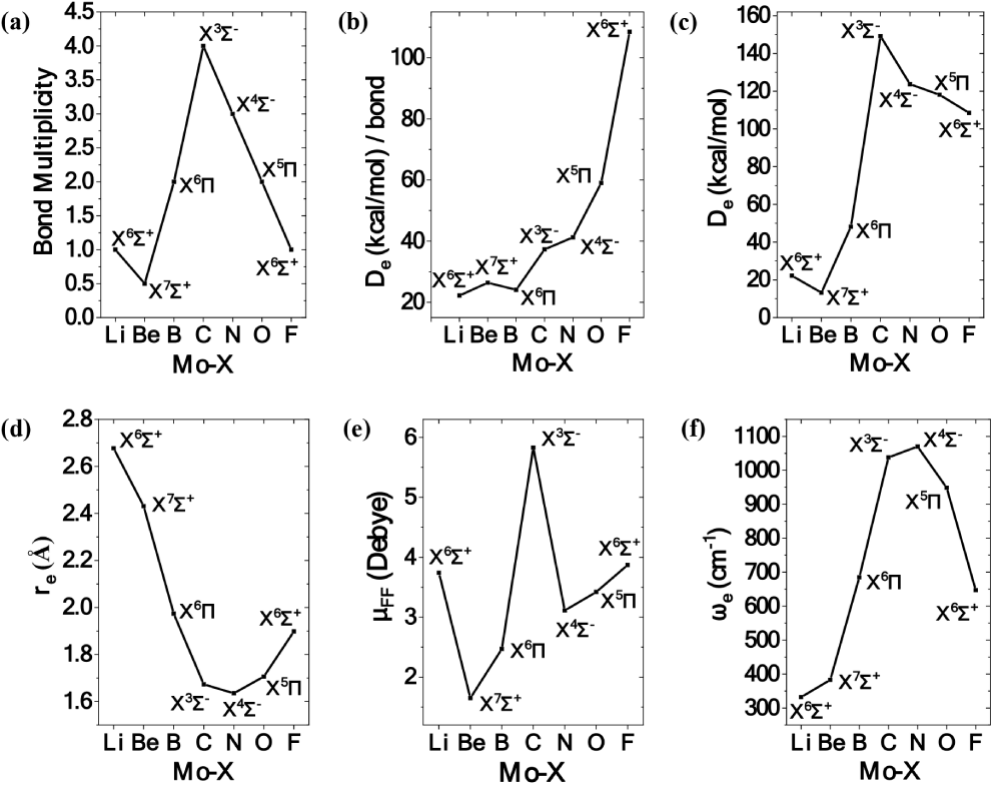
Bond multiplicity (a), dissociation energy per bond (b), dissociation energy (c), equilibrium distance (d), finite field dipole moment (e), and harmonic frequency (f) as a function of the X atom at the C-MRCISD+Q level.

It is quite interesting that the equilibrium distances obtained at the DFT level are practically identical to those obtained from the multireference and coupled-cluster calculations, see Figure 8 and Table 2. The largest deviation between these two sets of methods is observed in the case of MoLi, and more specifically, when the TPSSh functional is used. However, when it comes to the calculation of dissociation energy, some deviations are also present among these methods. In general, density functional theory overestimated dissociation energies with respect to C-RCCSD­(T) and C-MRCISD+Q values. The only exception to this trend is observed in the ground state of MoO. Significant discrepancies are observed for MoBe as well, since DFT overestimated dissociation energy by 5 to 10 kcal/mol (depending on the functional being used) with respect to our best C-RCCSD­(T) value of 14.4 kcal/mol. Moreover, for the ground states of MoLi, MoN, MoO, and MoF, MN15 overestimated dissociation energies by 5 to 10 kcal/mol with respect to our reference method [C-RCCSD­(T)]. Finally, comparing the three functionals employed in this work, it seems that overall, B3LYP shows a smaller deviation in the calculated properties than the MN15 and TPSSh functionals. Again, C-RCCSD­(T) represents our reference point for this comparison.

The bond multiplicity, dissociation energy, dissociation energy per bond, equilibrium distance, finite field dipole moment and harmonic frequency of the ground state of each MoX species are plotted in Figure 9 as a function of the X atom. It can be concluded that D_e_ is inversely proportional to r_e_, as an increase in dissociation energy corresponds to a decrease in equilibrium distance, and vice versa. Apart from the ground state, *X* ^3^Σ^–^, of MoC, and the ground state, *X* ^6^Σ^+^, of MoF, equilibrium products and asymptotic products are identical. In these two cases, equilibrium is dominated by an ionic picture, i.e., Mo^+^ + X^–^, since a strong charge transfer takes place from the metal to the X atom.

The present work highlights the exceptional ability of molybdenum atom to participate in a variety of bonding schemes, see Figure 9. To be more specific, in MoLi, one two-electron σ bond is formed; in MoBe, one one-electron σ bond (half bond); in MoB, one two-electron π, one one-electron π, and one one-electron σ bonds; in MoC, two two-electron σ, and two two-electron π bonds; in MoN, one two-electron σ, and two two-electron π bonds; in MoO, one two-electron σ, and one two-electron π bonds; and finally in MoF, one two-electron σ bond is formed. Overall, the ground states of MoX exhibit a diverse range of chemical bonds, from a half bond in MoBe to a quadruple bond in MoC, while all types of bonds are observed, i.e., dative, covalent, and ionic, see Figure 9a.

For the ground states, dissociation energies range from 14.4 (MoBe) to 149.2 (152.5; CBS limit) kcal/mol (MoC) with respect to adiabatic products. That is, MoBe is the least bound while MoC is the most bound system, see Figure 9c. To evaluate bond strength, the dissociation energy per bond as a function of X atom has been plotted. It turns out that D_e_/bond values are similar for MoLi, MoBe and MoB (∼25 kcal/mol), while as we move from C to F, D_e_/bond increases monotonically (MoC, MoN: ∼ 40 kcal/mol; MoO: 61 kcal/mol; MoF: 111 kcal/mol), see Figure 9b. The trend of the De_e_ values of Figure 9c is expected, and it is consistent with the number of the bonds that are formed. Thus, from MoLi to MoBe the bond multiplicity is decreased, and the D_e_ does the same. From MoBe to MoC the bond multiplicity is increased and also the D_e_ values do. Finally, from MoC to MoF the bond multiplicity is decreased and also the D_e_ values do. Finally, comparing the D_e_ values of molecules with the same bond multiplicity, i.e., for the pair of the MoO and MoB molecules that present a double bond, MoO presents the largest D_e_ value since the Mo–O distance is smaller than Mo–O distance due to the smaller atomic radius of O than B [56], see discussion below. Similarly, for the pair of MoF and MoLi that present a single bond, MoF presents the largest D_e_ value since the Mo–F distance is smaller than Mo–Li distance due to the fact that the MoF molecule is predominantly ionic and the F- anion has an increased bond radius.[56]

The graph that depicts the equilibrium distance as a function of the X atom has a “V” shape, see Figure 9d. The shortest bond distance is observed for MoN at 1.627Å, while the largest one is found for MoLi at 2.667 Å. That is, the latter distance is 1 Å larger than the former one. It is quite interesting that the bond length of MoBe is shorter than the one of MoLi, even though MoLi is a more bound system with a full σ bond. This trend is due to the reduction of the atomic radius from Li to F (167 ppm to 42 ppm).^66^ Specifically, from Li up to C the reduction of the atomic radius is sharp. From C to F, the reduction of the atomic radius becomes smaller. Thus, from MoLi to MoN the reduction of the Mo-X bond distance follows the reduction of the X atomic radius. Moreover, this reduction of the bond distance from MoBe to MoC is consistent with the increase of the number of the formed bonds. Comparing MoN and MoO, the Mo-X distance is increased from MoN to MoO even though the atomic radius of O is smaller, however, this is attributed to the fact that in MoN the bond is a triple one, while in MoO is a double one which results to the opposite effect, i.e., in a small increase of the Mo–O bond distance. Finally, the longer Mo–F bond distance compared to the Mo–O, Mo–N, and Mo–C bonds is attributed not only to the single-bond character of Mo–F, in contrast to the multiple-bond nature of Mo–O, Mo–N, and Mo–C, but also to the predominantly ionic character of the Mo–F bond and the larger ionic radius of the F^–^ anion.^67^

The dipole moments of MoX range from 1.24 D (MoBe) to 5.83 D (MoC), while the corresponding plot has a “W” shape. Large μ values are found for MoLi (3.73 D) and MoF (3.64 D), whereas it should be noted that the dipole moments of MoBe, MoB, MoN, MoO and MoF almost belong to the same straight line. A last comment regarding the calculation of dipole moment: Apart from MoLi and MoBe, where there is a considerable difference between the expectation values and the finite field values (the finite field values are larger by 1 D), both methods predict similar values for the rest of the molecules. In cases where two dipole moments differ significantly, the finite field method is in general more reliable [61].

The graph that depicts the harmonic frequencies as a function of the X atom has a “Λ” shape, see Figure 9f. The smallest harmonic frequency is observed for MoLi (300 cm^–1^) while the largest one is found for MoN (1093 cm^–1^). Finally, Table 5 gathers our best results, i.e., “recommended” bond distances, dissociation energies, dipole moments, equilibrium products, and asymptotic products.

**Table 5:** Best Calculated Values of Bond Distances r_e_ (Å), Adiabatic Dissociation Energies D_e_ (kcal/mol), Dipole Moments $\mu_{\mathrm{FF}}$ (D), Equilibrium Products and Asymptotic Products of the Ground States of MoX at the c-Mrcisd+q­[c-Rccsd­(t)]/aug-Cc-pwCv5z­(−pp) Computational Level

Species	State	r_e_	D_e_	μ_FF_	Equilibrium Products	Asymptotic Products
MoLi	X^6^Σ^+^	2.677[2.667]	22.2[24.4]	3.74[3.73]	Mo(a^7^S) + Li (^2^S)	Mo(a^7^S) + Li(^2^S)
MoBe	X^7^Σ^+^	2.431[2.452]	13.2[14.4]	1.65[1.24]	Mo(a^7^S) + Be (^1^S)	Mo(a^7^S) + Be(^1^S)
MoB	*X* ^6^Π	1.974[1.959]	48.2[47.2]	2.47[2.31]	Mo(a^7^S) + B (^2^P)	Mo(a^7^S) + B(^2^P)
MoC	X^3^Σ^–^	1.671^a^	152.5^a^	5.83	Mo^+^(a^6^S) + C^–^(^2^P)^b^	Mo(a^5^S) + C(^3^P)
MoN	*X* ^4^Σ^–^	1.635[1.627]	123.7[122.6]	3.11[3.09]	Mo(a^7^S) + N (^4^S)	Mo(a^7^S) + N(^4^S)
MoO	*X* ^5^Π	1.706[1.696]	118.1[122.9]	3.42[3.15]	Mo(a^7^S) + O (^3^P)	Mo(a^7^S) + O(^3^P)
MoF	X^6^Σ^+^	1.898[1.895]	108.5[111.4]	3.87[3.64]	Mo^+^(a^6^D) + F^–^(^1^S)	Mo(a^7^S) + F(^2^P)

a CBS limit at the C-MRCISD+Q level using the aug-cc-pwC*n*Z­(−PP) basis sets, *n* = 2–5; ref. 23

b Equilibrium is dominated by an ionic picture, i.e., ${\mathrm{Mo}}^{+}$ + $C^{-}$ . However, the equilibrium products could also be Mo (^7^S) + C (^5^S) via a significant charge transfer from Mo to C; ref. 23

## 4 Conclusion

The electronic structure and bonding of the ground and excited states of MoX species, where X = Li, Be, B, C, N, O, F, have been investigated employing multireference configuration interaction and single-reference coupled-cluster methodologies in conjunction with valence and weighted core valence correlation consistent basis sets of quintuple quality. Density functional theory calculations have also been performed in order to determine which functional most closely agrees with the multireference and coupled-cluster methods.

It is found that the DFT (B3LYP, MN15, TPSSh) geometries are in excellent agreement with the C-RCCSD­(T) and C-MRCISD+Q ones. On the contrary, regarding the dissociation energies, DFT overestimated dissociation energies with respect to C-RCCSD­(T) and C-MRCISD+Q values, except in the ground state of MoO. Moreover, significant discrepancies are observed for MoBe, which presents the smallest D_e_ values. Finally, comparing the three functionals employed in this work, it seems that overall, B3LYP shows a smaller deviation in the calculated properties than the MN15 and TPSSh functionals.

Furthermore, the chemical bonding of the MoX molecules was analyzed and characterized as dative, covalent, or ionic based on the leading CSFs, the population analysis and on the plot of the valence molecular orbitals and electron density.

The molybdenum atom has six unpaired electrons and can contribute all of them to the formation of chemical bonds. In our cases, the bonds formed range from a half bond (MoBe) to a quadruple bond (MoC), with all types of bonds being observed, including dative, covalent, and ionic. In particular, the following bonding schemes have been observed: MoLi (*X* ^6^Σ^+^): σ^2^, MoBe (*X* ^7^Π^+^): σ^1^, MoB (*X* ^6^Π): σ^1^π^2^π^1^, MoC (*X* ^3^Σ^–^): σ^2^σ^2^π^2^π^2^, MoN (*X* ^4^Σ^–^): σ^2^π^2^π^2^, MoO (*X* ^5^Π): σ^2^π^2^, and MoF (*X* ^6^Σ^+^): σ^2^. For MoC (*X* ^3^Σ^–^) and MoF (*X* ^6^Σ^+^), equilibrium is dominated by an ionic picture, i.e., MO^+^ + X^–^, since a strong charge transfer takes place from the metal to the X atom while avoided crossings are observed. For the remaining five molecules, the equilibrium and asymptotic products are identical.

For the ground states, dissociation energies range from 14.4 (MoBe) to 149.2 (152.5; CBS limit) kcal/mol (MoC) with respect to adiabatic products. That is, MoBe is the least bound while MoC is the most bound system, see Figure 9c. To evaluate bond strength, the dissociation energy per bond as a function of X atom has been plotted. It turns out that D_e_/bond values are similar for MoLi, MoBe and MoB (∼25 kcal/mol), while as we move from C to F, D_e_/bond increases monotonically (MoC, MoN: ∼ 40 kcal/mol; MoO: 61 kcal/mol; MoF: 111 kcal/mol). The trend of the De_e_ values is consistent with the number of the bonds that are formed, i.e., the increase of the bond multiplicity corresponds to an increase of the D_e_ value. For the pairs of molecules which have the same bond multiplicity, i.e., for the pair of the MoO and MoB molecules that present a double bond or for the pair of MoF and MoLi that present a single bond, the molecule with the shortest bond length has the largest D_e_ value.

The graph that depicts the equilibrium distance as a function of the X atom has a “V” shape, see Figure 9d. The shortest bond distance is observed for MoN at 1.627Å, while the largest one is found for MoLi at 2.667 Å. This shape is due to the reduction of the size of atomic radius from. Thus, from MoLi to MoN the reduction of the Mo-X bond distance follows the reduction of the X atomic radius and the increase of the number of the formed bonds. Comparing MoN and MoO, the Mo-X distance is increased from MoN to MoO even though the atomic radius of O is smaller, due to the fact that in MoN the bond is a triple one, while in MoO is a double one. Finally, the longer Mo–F bond distance, relative to the Mo–O, Mo–N, and Mo–C bonds, can be attributed not only to the single-bond character of Mo–F, unlike the multiple-bond character observed in Mo–O, Mo–N, and Mo–C, but also to the predominantly ionic nature of the Mo–F bond and the larger ionic radius of the F^–^ anion.^67^

The dipole moments of MoX range from 1.24 D (MoBe) to 5.83 D (MoC), while the corresponding plot has a “W” shape. Large μ values are found for MoLi (3.73 D) and MoF (3.64 D). Apart from MoLi and MoBe, where there is a considerable difference between the expectation values and the finite field values (the finite field values are larger by 1 D), both methods predict similar values for the rest of the molecules.

To sum up, the atomic or anionic radius and the atomic states of the involved atoms affect the bond distances, bonding, dissociation energies and other related properties of the diatomic molecules. Understanding how different atoms affect bonding and molecular properties is essential for rationalizing chemical trends, predicting reactivity, and designing new molecules and materials with desired functions. It bridges fundamental theory with real-world application. Thus, we hope that our contribution will be proven useful for both experimentalists and theoreticians, as it lays the foundation for deciphering the electronic structure “secrets” of complex transition metal compounds.

## #

Department of Chemistry and Biochemistry, Auburn University, Auburn, Alabama 36849, USA
